# Integrative structural modelling reveals the human Mic60-Mic19 subcomplex as a diffusion barrier in mitochondria

**DOI:** 10.1038/s41467-026-77869-3

**Published:** 2026-09-26

**Authors:** Evangelia Nathanail, Edoardo Rolando, Max Ruwolt, Iryna Zaporozhets, Fan Liu, Cecilia Clementi, Oliver Daumke

**Affiliations:** 1 Structural Biology, Max Delbrück Center for Molecular Medicine in the Helmholtz Association (MDC), Berlin, Germany; 2 Institute of Chemistry and Biochemistry, Freie Universität Berlin, Berlin, Germany; 3 Department of Physics, Freie Universität Berlin, Berlin, Germany; 4 Department of Structural Biology, Leibniz-Forschungsinstitut für Molekulare Pharmakologie (FMP), Berlin, Germany; 5 Charité-Universitätsmedizin Berlin, Berlin, Germany

**Keywords:** X-ray crystallography, Proteins, Computational models, Molecular conformation

## Abstract

Mitochondrial crista junctions (CJs) operate as regulated gateways into the cristae microenvironment, whose protein, metabolite, and ion compositions are finely tuned for mitochondrial function. The Mic60-Mic19 complex of the mitochondrial contact site and cristae organizing system (MICOS) complex was suggested to span across CJs and act as a diffusion barrier, but little is known of how its dynamic architecture facilitates this task. To address this question, we determine the crystal structure of an amino-terminal dimeric helical bundle of human Mic60. These and previous structural and biochemical data are harnessed in molecular dynamic (MD) simulations to develop a dynamic model of the human tetrameric Mic60-Mic19 subcomplex in the CJ environment, to validate its architecture using *in organello* and in vitro cross-linking data and to computationally characterize its function as a diffusion barrier. Our integrative structural biology approach enables the functional investigation of flexible, multidomain protein complexes which escape conventional structural methods.

## Abstract (web summary)

Mitochondrial crista junctions serve as entry points to cristae, with MICOS governing their form and composition. Here, authors resolve the dynamic structure of the human Mic60 subcomplex and how it spans across CJs to act as a diffusion barrier.

## Introduction

Mitochondria are highly dynamic organelles that serve as central hubs for energy production, amino acid and fatty acid metabolism, and the regulation of apoptosis in eukaryotic cells^1–3^. The morphology and plasticity of their network vary significantly across different organisms, tissues, and even within individual cells, reflecting the intricate relationship between their diverse cellular roles and their structure^4–6^. Mitochondria are characterized by their double membrane architecture^7^, comprised of a semi-permeable outer mitochondrial membrane (OMM) and an impermeable inner mitochondrial membrane (IMM). The IMM features a vastly extended surface area due to cristal invaginations that extend from the intermembrane space (IMS) into the matrix.

Cristae function as specialized microenvironments within the IMS and the IMM, where the oxidative phosphorylation machinery is located^8,9^. These regions are characterized by distinct protein and lipid compositions that coordinate the intricate cristal membrane architecture and optimize mitochondrial adenosine triphosphate (ATP) production^10–13^. ATP synthase dimer ribbons stabilize negative curvature at the cristal rim^14^, while Optic Atrophy 1 (OPA1) forms ring-like assemblies essential for maintaining the morphology of the cristal lumen^5,15–17^. A critical architectural component of cristae for establishing a distinct mitochondrial microenvironment is the crista junction (CJ), which acts as a diffusion barrier between cristae and the IMS, regulating the passage of proteins, metabolites, and ions^18,19^.

The mitochondrial contact site and cristae organizing system (MICOS) complex localizes to CJs and plays a crucial role in their formation and stability^20–22^. MICOS is a conserved multi-subunit protein complex of approximately 700 kDa in size and can be biochemically separated into the Mic10 subcomplex (containing Mic10, Mic26 and Mic27) and the Mic60 subcomplex (containing Mic60, Mic19 and its paralog Mic25 in vertebrates). The Mic12 subunit serves as a bridging component between the subcomplexes in yeast^23–26^, while its putative mammalian orthologue, Mic13, may not have such a function^27^. The Mic10 subcomplex is predominantly membrane-embedded and is thought to form a curved scaffold essential for stabilizing CJs^28–31^. In contrast, the Mic60 subcomplex is largely exposed to the IMS and anchored to the IMM by a single N-terminal transmembrane helix (TM) of Mic60. Deletion of either Mic10 or Mic60 results in a near-complete loss of CJs, with cristae forming lamellar sheet-like structures that are disconnected from the IMS^20–22^. In addition to shaping the IMM, the Mic60 subcomplex interacts with proteins of the OMM, such as the sorting and assembly machinery (SAM), bridging the intermembrane space and contributing to mitochondrial protein import and trafficking^32–36^.

Though MICOS is essential for regulating mitochondrial form and function, the molecular mechanism by which it stabilizes CJs remains poorly understood. First structural and biochemical insights on the Mic60 subcomplex were derived from thermophilic yeast orthologues. They revealed that Mic60 exists in a dimeric, autoinhibited state^37^, while Mic19 binding induces Mic60 tetramerization via a conserved interface in the coiled-coil (CC) domain^38^. The C-terminal mitofilin domain forms a domain-swapped dimer featuring a bent peripheral membrane binding site, which may bind to the highly curved CJ membrane. The Mic60-Mic19 subcomplex was suggested to span across the CJ and act as a mechanical strut preserving CJ diameter^38^.

While the current Mic60-Mic19 model was derived from fungal MICOS components, comparable data on the Mic60 subcomplex of higher eukaryotes have remained scarce. However, such data are crucial to understand the function of the Mic60-Mic19 complex in human mitochondria, particularly since both CJ morphology and Mic60 size vary between animals and fungi^39,40^. The lack of structural data for the human complex may be attributed to Mic60’s high structural flexibility and the intricate oligomerization network of the Mic60-Mic19 complex, which hinders structural characterization by conventional structural biology techniques. Recent efforts sought to investigate the structure of the subcomplex by AlphaFold predictions^41^ and to further validate these models through a subset of cross-links from human mitochondria^42^ or to characterize them in large-scale molecular dynamics simulations of mitochondrial cristae^43^. However, AlphaFold predictions of homo-oligomeric coiled-coils, such as the core of Mic60, are often problematic^44^. In addition, these models did not consider the hetero-octameric stoichiometry of the Mic60-Mic19 complex. Accordingly, the structural predictions remained incomplete, and the role of the Mic60-Mic19 subcomplex in the control of CJ architecture and function was not addressed in these studies.

Here, we present an integrative structural biology approach to model the human Mic60-Mic19 complex in its assembled form in a CJ-like environment, combining an experimental dimeric crystal structure of human Mic60 with in vitro, in silico, and *in organello* data. Experimentally solved and biochemically validated structures of nearly all folded regions of Mic60 across multiple organisms provide the foundation for this model, which is further refined through molecular dynamics simulations and validated by cross-linking mass spectrometry of intact mitochondria and of a recombinantly expressed Mic60 construct. The high dynamics of the Mic60-Mic19 complex facilitate its function as a diffusion barrier, as analyzed by coarse-grained molecular dynamics simulations. Our approach highlights the pivotal and multifaceted role of the Mic60-Mic19 subcomplex in maintaining CJ architecture and function and serves as a pipeline for studying dynamic multi-domain protein assemblies which have so far escaped structural characterization due to limitations of conventional structural biology methods.

## Results

### Human Mic60 features an N-terminal dimeric helical bundle

**Fig. 1:**
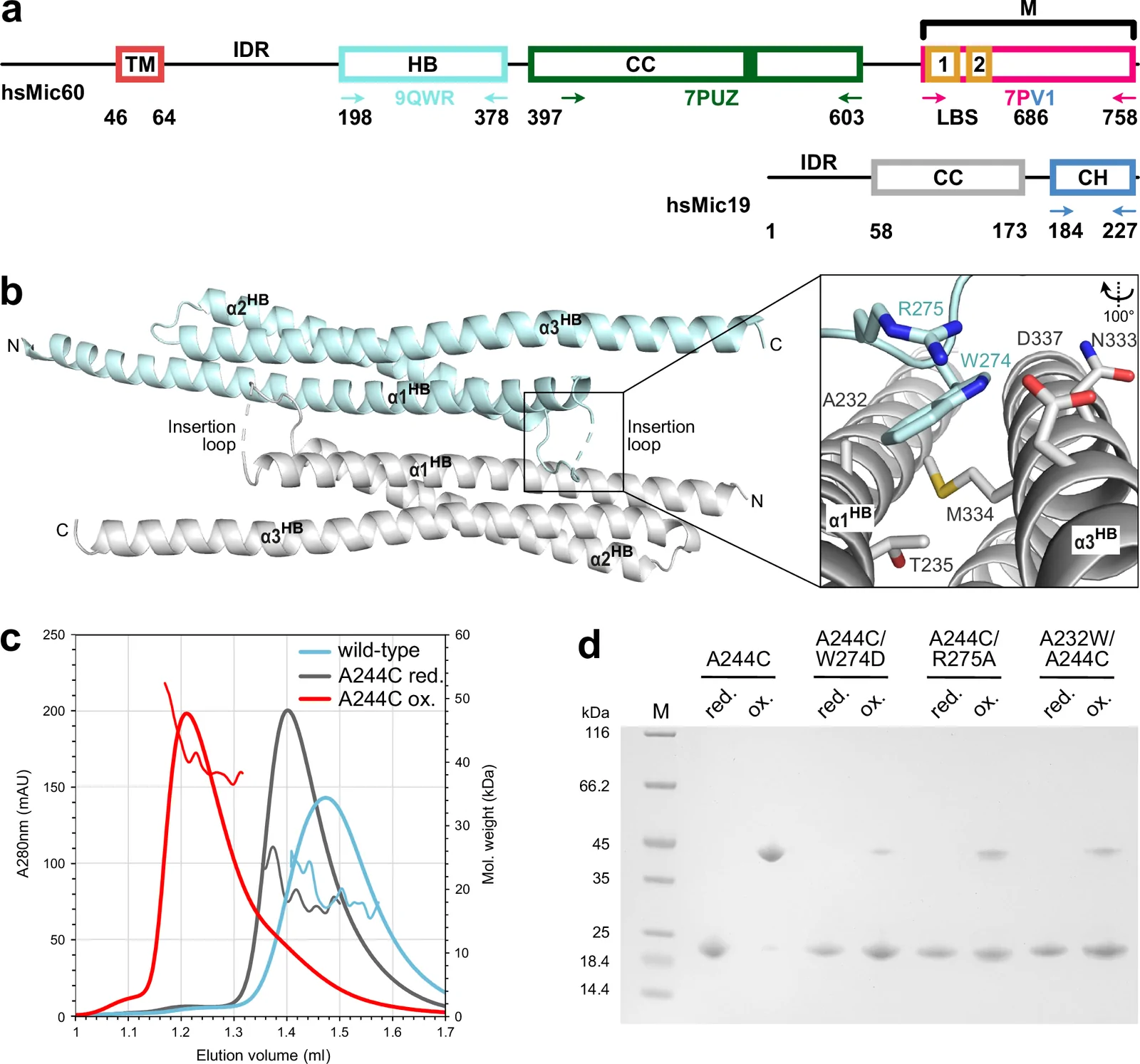
Animal Mic60 orthologues possess an N-terminal helical bundle with a conserved fold. **a** Domain architecture of hsMic60 and hsMic19, with residue numbers annotated below the domains. Solved orthologue crystal structures are indicated by arrows and PDB ID (helical bundle (HB) in cyan (PDB ID: 9QWR), coiled-coil (CC) in dark green (PDB ID: 7PUZ), mitofilin domain in pink and CHCH domain in blue (PDB ID: 7PV1)). **b** Cartoon representation of the hsMic60 N-terminal helical bundle (Mic60 HB). N- and C- termini of each monomer are labeled. Close-up view of the dimerization interface via an antiparallel loop insertion of residue W274, coordinated by R275, into an interface composed of helices α1^HB^ and α3^HB^ of the opposite moiety. **c** Size-exclusion chromatography and Right-Angle Light Scattering (SEC-RALS) profile of wt Mic60 HB (cyan) and the A244C variant in a reduced (gray) and oxidized (red) state captures transient dimer formation. **d** Non-reducing SDS PAGE of Mic60 HB cysteine mutants before and after CuSO_4_ oxidation (from left to right: A244C, A244C/W274D, A244C/R275A, A232W/A244C). The experiment was performed in triplicate and was quantified in Supplementary Fig. 3c.

While previous structural studies of MICOS were based on fungal Mic60 and Mic19 orthologues^38^, structural data on the human MICOS complex have remained limited. Animal Mic60 harbors a predicted helical bundle (HB) situated between the intrinsically disordered region (IDR) and the CC domain, which is not present in the fungal Mic60 counterparts (Fig. 1a and Supplementary Fig. 1). An evolutionary analysis based on the framework presented by Huynen et al.^45^ indicates that the Mic60 HB has emerged during animal evolution as the organisms acquired more complex multicellularity (Supplementary Fig. 1a).

We recombinantly expressed the HB domain of human Mic60 (homo sapiens (hs) Mic60, residues 198–378, hereafter referred to as Mic60 HB, Fig. 1a) and purified it to homogeneity (Supplementary Fig. 2a, b). Crystals of this construct diffracted to 2.78 Å resolution (2.95 Å with a more conservative resolution cut-off), and the structure was solved by molecular replacement using an AlphaFold prediction of the HB domain as a template (PDB ID: 9QWR, see Supplementary Table 1 for data collection and refinement statistics and Supplementary Fig. 2c for a representative electron density map).

The structure of the HB domain comprises an elongated three-helix bundle of helices α1^HB^-α3^HB^ (Fig. 1b). It features a hydrophobic, conserved core, pointing to stable domain architecture (Supplementary Fig. 1b). Interestingly, the two HB domain monomers in the asymmetric unit self-associated into a two-fold symmetric antiparallel dimer (Fig. 1b). Dimerization is mediated primarily by helix α1^HB^, with the interface spanning an area of 1060 Å^2^, stabilized by a symmetric loop insertion (Supplementary Fig. 3a). Residues W274 and R275 of the insertion loop embed into a hydrophobic groove composed of helices α1^HB^ and α3^HB^ of the opposite Mic60 HB monomer. Notably, W274 and R275 are surface-exposed and highly conserved in vertebrate Mic60 (Supplementary Fig. 1b, c), pointing to their functional importance.

We characterized the assembly status of the Mic60 HB by size-exclusion chromatography coupled to right angle laser scattering (SEC-RALS), in which the protein eluted as a monomer (Fig. 1c). To identify a possible transient dimer, we engineered a structure-based disulfide bond by introducing the A244C mutation in the center of the hydrophobic dimerization interface (Supplementary Fig. 3a). Indeed, Mic60 HB A244C predominantly formed dimers under oxidative conditions (Fig. 1c). When the disulfide-stabilizing variant was combined with mutations either within the loop (W274D, R275A) or in the hydrophobic groove of the opposite monomer (A232W), the combinatorial mutants showed markedly reduced dimer formation. These data agree with a model indicating that W274 and R275 mediate transient dimerization of Mic60 HB (Fig. 1d and Supplementary Fig. 3b, c). Accordingly, the HB domain may act as a further oligomerization domain in animal Mic60.

Despite low sequence similarity, a FoldSeek^46^ homology search revealed that the Mic60 HB possessed the highest structural similarity to the IMS protein Smac/DIABLO, which is a known apoptosis regulator^47^ (Supplementary Fig. 3d). Like Mic60 HB, Smac/DIABLO also forms a dimer; however, it employs a distinct dimerization interface and lacks a structural equivalent to the insertion loop observed in Mic60 HB (Supplementary Fig. 3e). These observations indicate an unexpected evolutionary relation of the MICOS complex and apoptosis regulators in animalia.

### Assembling a homology-based structural model of the human Mic60-Mic19 complex

Three crystal structures cover the assembly of nearly all folded regions of Mic60: The dimeric Mic60 HB domain presented here, the tetrameric CC domain of *Lachancea thermotolerans* Mic60 (PDB ID: 7PUZ), and a fusion construct containing the dimeric Mic60 mitofilin domain and the Mic19 CHCH domain from *Chaetomium thermophilum* (PDB ID: 7PV1). Building on these structures, we constructed a homology-based model of the human Mic60-Mic19 hetero-octameric complex. As structural prediction algorithms failed to accurately reproduce the known biochemical interactions, we assembled the complex manually. To this end, human homology models of the tetrameric coiled-coil region and the mitofilin domain were generated using SWISS-MODEL^48^ and combined with AlphaFold predictions of disordered or loop regions to construct a tetrameric model of human Mic60^41^. The CHCH domain of human Mic19 was positioned on the human Mic60 mitofilin region based on the previously published structure of the *Chaetomium thermophilum* complex, while the remaining Mic19 regions were excluded from the model due to ambiguous conformations in AlphaFold predictions. To characterize the assembly of the human Mic60 CC domain, we employed blue native gel polyacrylamide (BN-PAGE) and SEC-RALS analysis of two CC constructs with different C-terminal truncations. Consistent with results previously obtained for *Lachancea thermotolerans* Mic60 CC, the human Mic60 CC domain appeared in dimeric or tetrameric forms, where the assembly was controlled by its adjacent regions (Supplementary Fig. 4a, b).

**Fig. 2:**
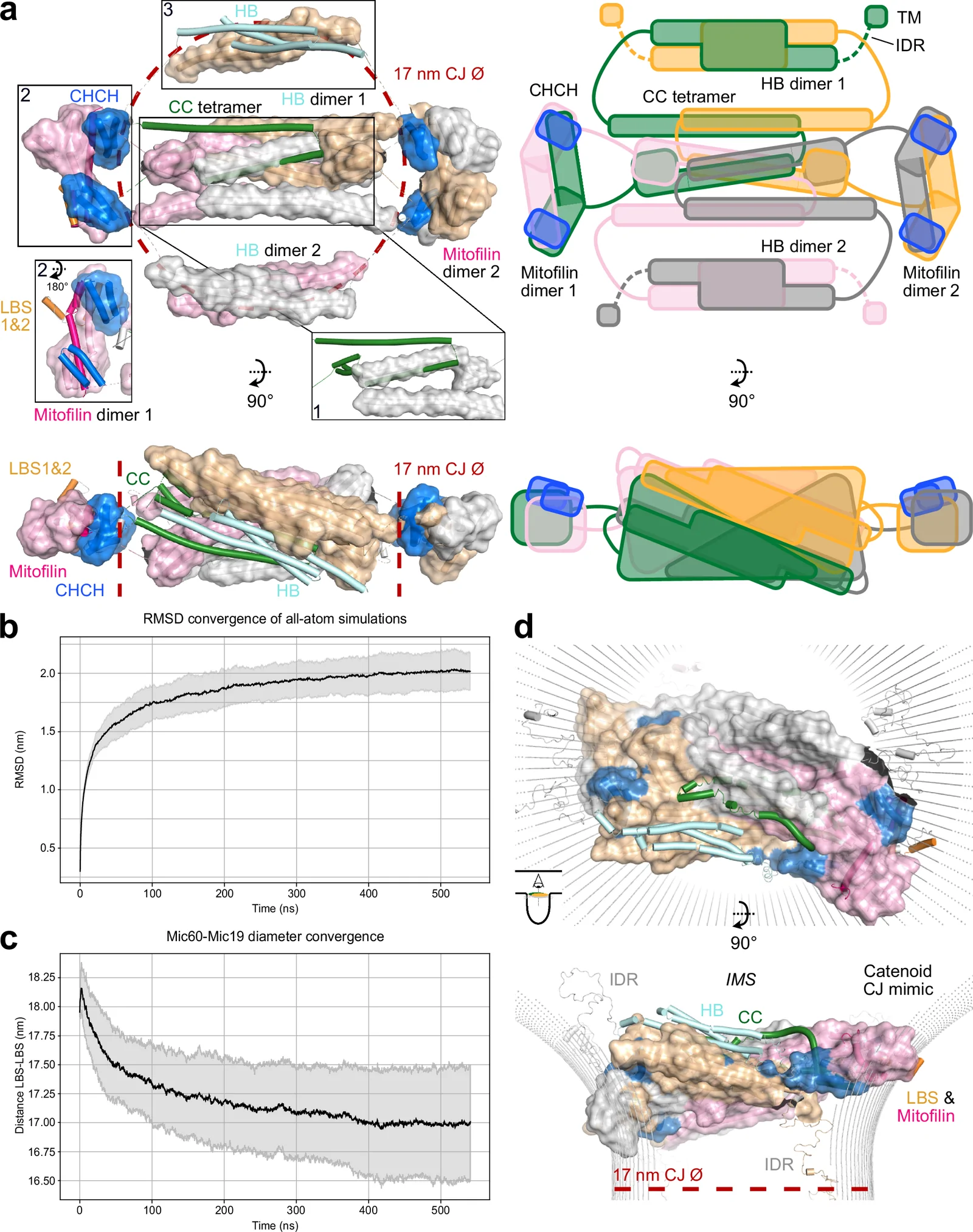
MD simulations of a homology-based structural model of the human Mic60-Mic19 complex on a CJ mimic. **a** Homology-based model of the human Mic60-Mic19 complex based on known crystal structures, displayed as cylinders and surface. Mic60 monomer one is colored according to annotated domains in Fig. 1a, monomers two, three, and four in sand, gray, and pink, respectively. The IDR is not displayed for illustration purposes. For Mic19, only the CHCH domains could be modeled confidently, shown here in blue. Known oligomerization domains of Mic60 are displayed in rectangle 1 (tetrameric CC, here only one dimer displayed for illustration purposes, PDB ID: 7PUZ, monomer 1 in forest green), 2 (mitofilin dimer, PDB ID: 7PV1, monomer 1 in orange and hot pink) and 3 (dimeric Mic60 HB, monomer 1 in cyan). Dashed lines indicate the width of a human CJ (17 nm) and the perpendicular placement of the complex in the CJ. A graphical sketch of the model is presented on the right, with monomer 1 colored in green and other monomers colored as in a (sand, gray and pink). **b** Root-Mean-Square deviation (RMSD) mean and standard deviation from the 30 all-atom simulations is shown over the time course of the simulation. **c** Diameter convergence of the human Mic60-Mic19 complex, shown as mean and standard deviation, during the 30 all-atom simulations, prior to the attachment to the catenoid. **d** Final snapshots of the Cα structure-based MD simulations of the homology-based model represented as cylinders and surface. The base diameter of the catenoid, indicated in a red dashed line, is 17 nm.

The assembled human Mic60-Mic19 model aligns with prior biochemical and structural observations and centers around a tetrameric CC core (Fig. 2a, rectangle 1). Importantly, the tetrameric CC supports the formation of the two C-terminal dimeric mitofilin-CHCH modules, which were described to form a highly curved membrane-binding region (Fig. 2a, rectangle 2)^38^. Surrounding the core, the Mic60 HB dimers and the highly flexible IDR occupy the remaining space (Fig. 2a, rectangle 3; IDR not shown). Short inter-domain linkers result in a mostly planar configuration of the complex, oriented perpendicularly to the CJ neck.

Due to missing structural data describing the interactions between the folded domains within the Mic60-Mic19 complex, linkers were initially added in an extended conformation to prevent domain clashes. As a result, the initial homology-based model adopted an overstretched, energetically strained conformation, with a lateral width exceeding the average CJ diameter of 17 nm observed in human mitochondria^49,50^ (see red dotted line in Fig. 2a).

As we could initially not determine how the HB dimers are positioned relative to the tetrameric CC, we also generated an alternative model which was significantly more extended (Supplementary Fig. 4c, d). Further *in organello* validation (see below) supported the first, more compact model on which the following computational analyses were focused.

### Molecular dynamics simulations reveal the dynamic conformation of the human Mic60-Mic19 complex

To relax the unfavorable conformation and obtain dynamic information of the Mic60 IDRs in particular, we used the generated homology model as a starting point for molecular dynamics (MD) simulations. Domains were stabilized to reflect their assembly in the crystals, while no constraints were imposed on additional inter-domain contacts or unstructured regions.

Within 500 ns of initial all-atom MD simulations, an equilibration state was reached, as indicated by root-mean-square deviation (RMSD) convergence (Fig. 2b, Supplementary Fig. 5a). Notably, the simulations led to a compression of the structure along its longitudinal size, ultimately converging to an average width of 17 nm, as measured between the LBS regions (Fig. 2c and Supplementary Fig. 5b), which is consistent with previously reported diameters of human CJs^49,50^. Notably, the alternative model retained a higher average equilibrium width (Supplementary Fig. 5c, d).

The initial main model was used in parallel to initiate machine-learned coarse-grained (MLCG) simulations in order to explore faster conformational dynamics^51^. The MLCG simulations accordingly exhibited faster convergence compared to the all-atom simulations (Supplementary Fig. 6a) while retaining a similar average secondary structure content and overall architecture, revealing a consistent fold for the MD model of the Mic60-Mic19 complex (Supplementary Fig. 6b).

To characterize the membrane interaction of the Mic60-Mic19 complex, we performed coarse-grained MD simulations of the mitofilin-CHCH dimer using the Martini 3 force field, embedding it in a lipid bilayer with composition and curvature representative of the IMM environment. The mitofilin domain strongly bound to this membrane, with a substantial free energy gain of approximately 100 kJ/mol (Supplementary Fig. 7a, b). This supports a model in which the mitofilin domain dimer attaches with high affinity to the IMM.

To integrate the structural model into an environment mimicking the CJ architecture, we attached the relaxed all-atom Mic60-Mic19 models to a catenoid surface with a base diameter of 17 nm, using the first residue following the transmembrane domain (Ile62) and the LBS2 in the mitofilin domain as surface attachment points (Supplementary Fig. 7c, d). The membrane interaction strength was modeled to reflect the ~100 kJ/mol binding free energy observed in the coarse-grained simulations of the mitofilin-CHCH dimer. Although the all-atom model was free to move along the catenoid and, consequently, adapt to its width, it maintained an average width of 17 nm, consistent with the value reached during the prior unconstrained all-atom equilibration (Fig. 2c).

Both the all-atom force field and the MLCG model led to an artificial compression of the IDRs, as had been previously observed when simulating large IDR assemblies^52–54^ (Supplementary Fig. 7c, d). To more accurately capture IDR dynamics, the final frames from each of the 30 all-atom runs were converted into coarse-grained Cα structure-based models^55^, leading to a refined final structure (Fig. 2d). Overall, the MD simulations revealed a more compact organization of the inter-domain linker regions, with the dimeric Mic60 HB collapsing onto the tetrameric CC on both sides. AlphaFold2 predicted an extended helical connection between the Mic60 HB and the CC (see Materials and Methods), which consistently became sandwiched between these domains during the simulations (Supplementary Fig. 7e). In this way, a compact core structure was formed that covered almost the entire CJ pore. The folded core buried more than 3.000 Å^2^ and the assembly interfaces featured mainly hydrophobic interactions framed by salt bridges between conserved residues of α2^HB^ and the CC helical connection (Supplementary Fig. 7f). Accordingly, the HB-CC interaction remained stable throughout the simulation. In contrast, the IDRs were flexible in the coarse-grained Cα structure-based simulations, covering diverse trajectories within the remaining CJ space. In this way, the IDRs formed an extended network above and below the HB-CC core.

**Fig. 3:**
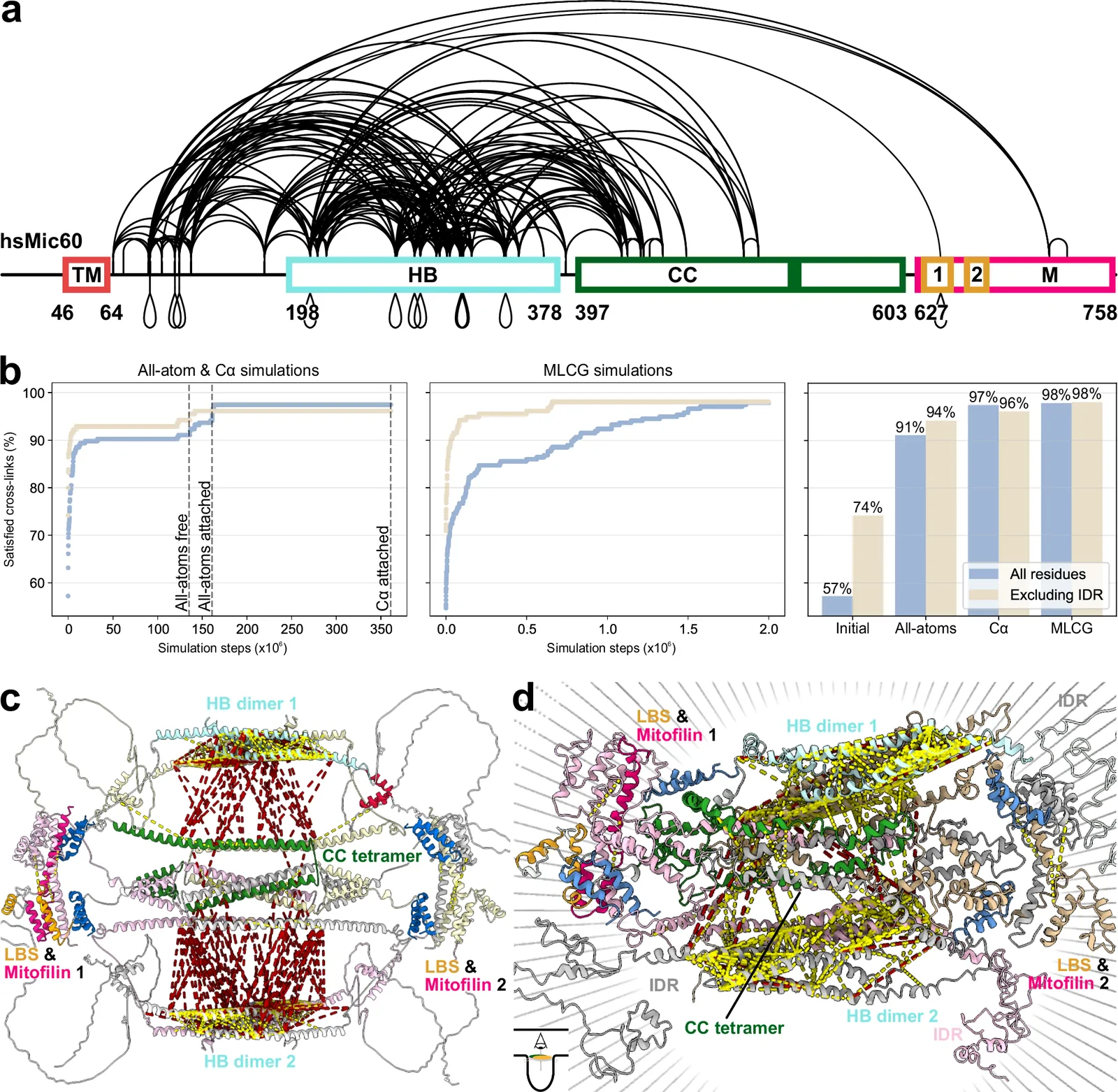
*In organello* cross-linking as model validation. **a** Visualization of the DSBSO mitochondrial cross-linking dataset on the Mic60 sequence, modified from xiNET^109^. **b** The percentage of cross-links satisfied across the frames of the simulation, shown for the all-atom and Cα pipeline (left) and the MLCG model (center). The MD simulations were separated into the initial equilibration of the all-atom simulation prior to attachment to the crista (“All-atoms free”, first 135 million steps), the equilibration of the all-atom model attached to crista (“All-atoms attached”, 25 million steps), and the Cα-based simulations attached to the crista (“Cα attached”, 200 million steps). Many of the cross-links involve the flexible IDR and were excluded in a second analysis (“Excluding IDR”). The rightmost panel shows the percentage of cross-links from the published DSBSO dataset observed in the human Mic60-Mic19 model before (“Initial”), after the all-atom simulations (“All-atoms”), after the subsequent Cα simulations (“Cα”), and using the MLCG model. Residue-residue cross-links were considered satisfied when the two residues of any chain fulfilled the distance cut-off for at least 1% of frames at any time up to the indicated time point in any of the parallel simulations. **c**,**d** The homology-based model of the human Mic60-Mic19 complex shown before (**c**, as in Fig. 2a) and after (**d**, as in Fig. 2d) the MD simulations. The cross-links annotated in a are visualized as dashed lines connecting the corresponding lysine residues (yellow for in range, red for out of range, cut-off set to 40 Å). All cross-links were visualized on the Mic60-Mic19 model using ChimeraX with the XMAS tool^92,93^.

To validate our MD simulations and obtain information on the Mic60-Mic19 structure in an organellar context, we utilized a publicly available human mitochondrial cross-linking dataset employing the enrichable and MS-cleavable cross-linker disuccinimidyl bis-sulfoxide (DSBSO)^56^. The database contains 236 unique residue pairs for hsMic60 distributed across the sequence (Fig. 3a, Supplementary Fig. 8, see also Source data for a detailed overview). These data were used to assess the ensemble of generated structures by quantifying the number of satisfied cross-links. In these analyses, distance constraints of 40 Å between the Cα of two lysines were considered, imposed by the spacer arm length of the DSBSO cross-linker while accounting for some degrees of domain and side-chain flexibility^56^. Notably, 57% of the observed cross-links were compatible with the initial model before the MD simulations, with the alignment rising to 74% when the flexible IDR was excluded from the analysis (Fig. 3b, c and Supplementary Fig. 9a). Compatible cross-links were primarily observed within the domains, while intra-domain cross-links remained out of range due to the extended domain-linker conformations (Fig. 3c, yellow for in range, red for out of range). Multiple valid inter-domain cross-links were detected between residues brought in proximity by domain homo-oligomerization, thereby confirming the assemblies observed in the crystal structures (Supplementary Fig. 9b). After the MD simulations, the structural model and the experimentally derived cross-links agreed further, as nearly all identified cross-links (97%) were satisfied at some time points during the MD simulations (Fig. 3b, d). Notably, in both scenarios, meaning whether all cross-links were considered or those involving the IDR were excluded, MLCG simulations showed improved agreement with the experimental data at shorter simulation times compared to all-atom simulations and a similar agreement to the Cα-based structural simulations, differing by only net one cross-link (Fig. 3b and Supplementary Fig. 6c).

To probe the data with a more stringent lysine-to-lysine distance, we compared the DSBSO-based dataset with a disuccinimidyl sulfoxide (DSSO)-based dataset from the same publication^56^. DSSO is a shorter crosslinker compared to DSBSO and, accordingly, allows for a shorter, more stringent distance cut-off of 35 Å. However, DSSO yields fewer and less confident cross-links compared to DSBSO as it is not enrichable (see Source data). The comparison revealed that the majority of DSSO residue-residue cross-links were recapitulated in the DSBSO dataset (Supplementary Fig. 10a). Furthermore, the DSSO-based residue-residue cross-links show a high satisfaction rate of 94% across the MD simulations, which is consistent with our DSBSO analysis even under more stringent cross-linking distances (Supplementary Fig. 10b).

To further validate the *in organello* cross-linking data and achieve higher spatial resolution, we recombinantly expressed a construct of the Mic60 HB and CC (hsMic60 residues 198-542, hereafter referred to as Mic60 HB-CC), which encompasses the majority of the cross-linked lysine residue pairs of the DSBSO dataset (65% for all, 99% excluding the IDR). We performed in vitro cross-linking using the short-armed cross-linker PhoX^57^ and observed that the same overall pattern of the DSBSO cross-links, in particular the bridging of the HB to the CC, was recapitulated in the purified construct (Supplementary Fig. 10c). Following the same procedure as for the *in organello* cross-links, we observed that 91% of the in vitro cross-links were satisfied at some point of the MD simulations, reaching up to 99% in the MLCG simulations, even using the more stringent cut-off of 30 Å of PhoX (Supplementary Fig. 10d). These in vitro experiments complement the *in organello* studies by validating the HB-CC packing in the core of our oligomeric model.

The overall increase in agreement after the MD simulations compared to the initial model was attributed, on the one hand, to the collapse of the HB on the CC core during the all-atom simulations, rendering multiple intra-domain cross-links valid. On the other hand, the Cα-based simulations reflecting the dynamic conformations of the IDR further improved the fit of the model to the cross-linking data (Fig. 3b, d). Thus, the *in organello* cross-linking results further support the notion of a highly dynamic model, in which the dimeric HB tightly packs against the Mic60 CC tetrameric core, with the flexible IDR surrounding them. The cross-linking validation of HB-CC packing favors the model presented here over the alternative model, which shows only 72% satisfied cross-links after all-atom simulations (Supplementary Fig. 5e, f).

### The Mic60 subcomplex as a diffusion barrier in human mitochondria

**Fig. 4:**
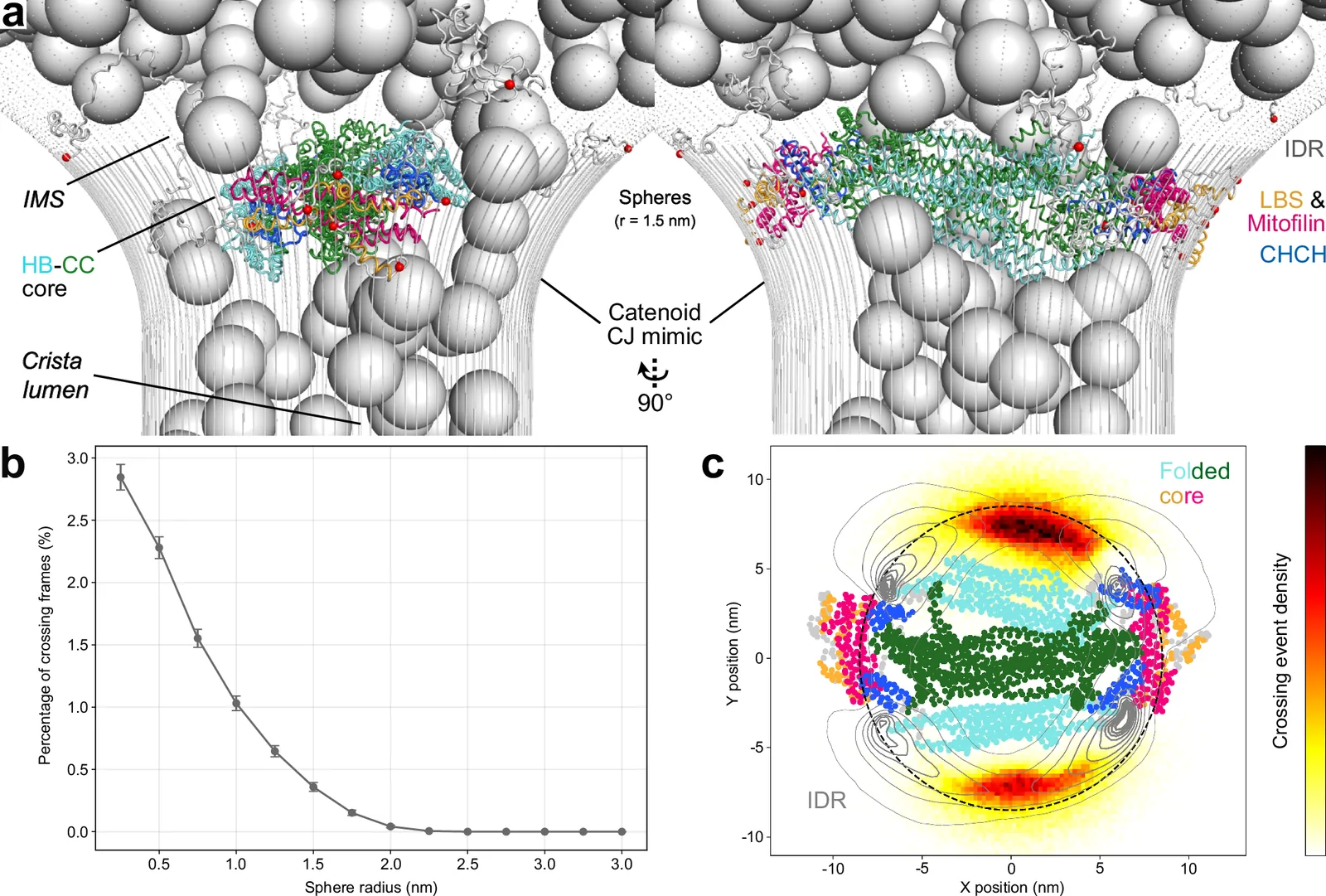
The human Mic60-Mic19 complex as a diffusion barrier in CJs. **a** Snapshot of the Cα-based diffusion experiments of the human Mic60-Mic19 model in the catenoid. 200 spheres, here with the suppression cut-off radius of 1.5 nm, were allowed to pass through the junction. There were no repulsion forces between the spheres, only between the spheres and the complex or the catenoid, allowing them to be simulated simultaneously. **b** Fraction of frames exhibiting an above-to-below Mic60-plane transition at equilibrium, normalized by the total number of frames, plotted as a function of sphere radius. Gray bars indicate the mean and standard deviation across all spheres (*n* = 200 independent spheres, shown as mean ± standard deviation and trend line). **c** Heat map depicting the density of sphere trajectories (radius 1.5 nm) projected onto the xy-plane during crossing events through the CJ in the presence of the human Mic60-Mic19 complex. Regions in black indicate the highest crossing density, while white indicates the lowest. The position of the complex throughout each simulation frame is projected onto the CJ plane. The average structure of the folded domains is displayed as a cartoon, while the IDR is illustrated as gray contour lines to better illustrate its movement. The dashed black line indicates the CJ diameter of 17 nm.

CJs were suggested to function as a diffusion barrier that regulates the exchange of proteins and metabolites between the cristae lumen and the IMS^18,58^. While the Mic60 subcomplex is the largest assembly with a soluble moiety in the CJ pore, direct experimental evidence supporting a role as a diffusion barrier has remained elusive. To investigate this idea computationally, we leveraged our dynamic model of the human Mic60-Mic19 complex to probe its capacity to restrict molecular diffusion. In these simulations, we assessed the ability of spherical particles of varying radii to traverse the CJ plane from the IMS into the cristae lumen (Fig. 4a and Supplementary Video 1). The MD simulations revealed a steady decrease in the flux of particles crossing the Mic60-Mic19-filled protein pore with increasing particle size. Crossing was partly suppressed for particles with a radius of 1.5 nm and was efficiently excluded for particles with a radius larger than 2 nm (corresponding to a globular protein of ~27 kDa^59^, Fig. 4b).

We previously suggested that the IDR of Mic60, a common feature across Mic60 orthologues, may play a role in impeding the passage of larger molecules^38^. To test this hypothesis, we analyzed a heat map of crossing events for particles at the established 1.5 nm suppression cut-off, stratifying the complex into two functional regions: the folded core (displayed as a colored cartoon) and the IDR (gray contour lines) (Fig. 4c). Projection of these regions across all simulation frames demonstrated that the dynamic movement of the IDR enables it to intermittently occupy wider sections of the catenoid-shaped membrane, thereby extending its spatial coverage beyond that of the structured core. As a result, the IDR effectively limits access through the CJ plane. When passing the IDR, particle passage was confined to the narrow strait between the folded complex core and the CJ membrane, severely restricting diffusion. In summary, our simulations suggest that the dynamic nature of the Mic60-Mic19 subcomplex and particularly the dynamics of the IDRs facilitate its function as a diffusion barrier.

## Discussion

Our study provides structural insight into the highly dynamic architecture of the human Mic60-Mic19 subcomplex through an integrative structural biology approach combining in vitro, in silico, and *in organello* data. Our MD simulations further indicate that the dynamic nature of the Mic60-Mic19 subcomplex is an important feature for its function as a mitochondrial diffusion barrier.

We identified the Mic60 HB as a previously uncharacterized oligomerization domain specific to animal orthologues, thus completing the structural annotation of the folded domains in human Mic60. Notably, the Mic60 HB forms an antiparallel dimer composed of three-helix bundles, reminiscent of the architecture adopted by the apoptotic signaling protein Smac/DIABLO^46,47^. Smac/DIABLO localizes to the IMS in non-apoptotic conditions and was identified as a MICOS interactor in the MINDNet network^60,61^, suggesting that this fold may represent a conserved structural domain in IMS proteins and function as an interaction platform.

A direct physiological role of the Mic60 HB in establishing CJ architecture is supported by observations in cardiomyocytes, which express a Mic60 splice variant lacking exon 6, corresponding to a deletion of residues 176–208, which include the N-terminal part of the HB^62^. While the missing residues do not directly contribute to HB dimerization based on our structure, the deletion could disrupt interactions with adjacent IDR residues or promote local unfolding and could therefore be linked to mitochondrial phenotypes observed in these cells, including elongated morphology and densely packed cristae^63,64^.

To fully leverage the available structural and biochemical information, we manually constructed an initial model of the human Mic60-Mic19 complex using experimentally determined crystal structures as a basis. Each domain was assumed and modeled to form homo-oligomers consistent with crystallographic and biochemical data: a HB dimer, a CC tetramer, and a mitofilin dimer. While both HB dimerization and CC tetramerization were experimentally addressed for the human orthologue, expression of the isolated human mitofilin domain was not successful, precluding similar biochemical validation. However, *in organello* cross-links from purified human mitochondria supported the proposed homo-oligomerization of all three domains, in agreement with the corresponding crystal structures of fungal homologs (Supplementary Fig. 9b). We further assumed that the human Mic60-Mic19 complex spans across the CJ neck, rather than spanning from the IMM to the OMM. A published computational model proposed that Mic60 functions as a monomer, anchored to the IMM via its TM domain and extending across the IMS to interact with the flat OMM with its mitofilin domain^43^. Contrasting this idea, the symmetric architecture required by Mic60’s antiparallel tetrameric state and the inclusion of Mic60’s TM domain in the IMS are not compatible with a monomeric, asymmetric IMM-OMM bridging role. Furthermore, our in silico analysis indicates that the mitofilin dimer exhibits strong binding to a curved membrane surface mimicking the IMM. In line with the model suggested for the fungal MICOS orthologues^38^, we therefore propose that the Mic60-Mic19 complex adopts a CJ-spanning architecture, with the tetrameric HB-CC core occupying the CJ pore and the mitofilin domains contacting the IMM via their lipid binding sites. The HB dimer, in the context of the fully assembled complex, significantly increases the CJ interface occupied by the folded complex by packing against the tetrameric CC, thereby also contributing to restricting diffusion in the tubular CJs of animalia.

A recent preprint^65^ reported that re-expression of a Mic60 HB deletion variant in Mic60 knockout mouse embryonic fibroblasts restored assembly of the MICOS complex and rescued the Mic60 deletion phenotype in respiration and ATP production, suggesting that the HB domain is largely dispensable for these functions in fibroblasts; however, in the rescued cells, fragmentation of the mitochondrial network was observed while cristae morphology remained mostly intact^65^. The latter finding is consistent with our evolutionary observation that the HB is not found in all MICOS- and CJ-possessing organisms. Instead, the HB may have distinct or cell-type-specific mitochondrial functions, for example, to enlarge the volume occupied by the Mic60 CC in certain CJ morphologies or stress conditions. In particular, the packing of the HB onto the CC appears to be vital for Mic60 function in neurons, as residue Lys299 of the HB constitutes part of the HB-CC interface (Supplementary Fig. 7f7, while the K299E mutation (895 A > G) has been associated with optic neuropathy and developmental encephalopathy due to altered mitochondrial membrane architecture in human patients^66^.

According to our integrative model, the Mic60-Mic19 hetero-octamer spans across the CJ at a diameter of 17–21 nm, with the flexible IDRs of Mic60 moving extensively in the CJ opening. Our diffusion simulations suggest the exclusion of proteins with a hydrodynamic radius of ~2 nm ( ~ 27 kDa). This would exclude larger assemblies like small TIM heterooligomers from crossing the CJ, but not smaller globular proteins such as cytochrome c, which are confined to the cristae^67,68^. It can be envisaged that the inclusion of further MICOS components into our model, most vitally the remaining folded domains of Mic19 or the membrane-embedded Mic10 subcomplex, will further restrict the size of particles that can cross the CJ pore. Furthermore, the Mic19 N-terminus has been proposed to reach over from the IMM to the OMM and contact the SAM complex^32^, forming a flexible network contact site through dynamic movement, which could be included in future modeling studies. We furthermore show that the flexibility of the IDR is a prerequisite for its function as a diffusion barrier through the crista junction. In our current simulations, the interaction of the IDR with the particles was repulsive. In contrast, the IDR may contribute to cargo selectivity via low-affinity interactions with the substrates. In an analogous way, phenylalanine-glycine repeats of the nuclear pore complex have been shown to bind to the human immunodeficiency virus, allowing its efficient translocation into the nucleus^69^.

Experimental structure solution of the entire MICOS complex, for example, by single-particle cryo-electron microscopy or in situ by cryo-electron tomography, is challenging due to its relatively small yet highly flexible domain architecture, the complex’s low copy number in CJs^70^, and the crowded CJ environment^71^. Compared to more static structures, the dynamic Mic60-Mic19 model proposed in this study rationalizes these challenges and may provide a more realistic structural representation required to understand MICOS function. Our model could also be integrated into larger-scale simulations of IMM protein organization, a challenge noted in previous efforts to simulate MICOS due to its inherent complexity and flexibility^43^. The applied integrative approach may provide a valuable benchmarking strategy for modeling proteins with IDRs, which pose a persistent challenge in structural biology due to their conformational heterogeneity. IDRs frequently mediate transient interactions and contribute to structural flexibility – features especially relevant in dynamic, membrane-associated assemblies that regulate diffusion, such as the nuclear pore^72^. Our approach builds on these insights by validating such simulations with *in organello* data, a concern often raised about in silico measurements^73^. By integrating experimental constraints with flexible modeling, we offer a framework for capturing the structural and functional contributions of disordered regions within large macromolecular complexes, while physiological validation anchors the model in a relevant cellular context. Our integrative human Mic60-Mic19 model therefore sheds light on the dynamic role of MICOS as a governor of CJ dynamics through a pipeline combining structural, biochemical, computational, and cross-linking data.

## Methods

### Cloning and plasmids

hsMic60 (UniProt ID: Q16891, codon-optimized cDNA synthesized by Eurofins Genomics) was used for cloning. The hsMic60 helical bundle (Mic60 HB, residues 198-378) was cloned via restriction sites *Nde*I and *Xho*I and subsequent ligation into the pET SUMO vector encoding an N-terminal His_6_-SUMO tag fusion protein. Mutations were introduced via site-directed mutagenesis^74^. The hsMic60 coiled-coil (hsMic60 CC, residues 473–542 and residues 473–617) and the HB-CC (hsMic60 HB-CC, residues 198–542) were cloned into the pET21b(+) vector encoding a human rhinovirus 3 C (HRV-3C) cleavable N-terminal MBP fused to a pMal_c2X linker using Gibson assembly.

### Expression and purification

Expression plasmids were transformed into BL21(DE3) *Escherichia coli* cells and plated on ampicillin-containing LB plates. Transformed bacteria were grown in terrific broth medium supplemented with 100 μg/ml ampicillin at 37 °C and 80 rpm until they reached an OD_600_ of 0.8 then cooled to 18 °C, and protein expression was induced with 300 μM isopropyl-ß-D-1-thiogalactopyranoside (IPTG) overnight. Cells were harvested via centrifugation for 20 min at 4,000 g and cell pellets were stored at −20 °C.

Mic60 HB pellets were thawed and resuspended in lysis buffer (20 mM 4-(2-hydroxyethyl)−1-piperazineethanesulfonic acid (HEPES)/NaOH pH 7.5, 500 mM NaCl, 20 mM imidazole), supplemented with 500 μM 4-(2-aminoethyl)-benzolsulfonyfluorid hydrochloride (AEBSF, BioChemica) and 1 μM DNase (Roche). Cells were lysed through sonication (5 × 45 s with 1 s pulses at 70% power), and lysates were centrifuged at 40,000 g for 45 min. The supernatant was filtered using a 0.45 μM filter and applied to Ni-NTA resin (Cytiva), equilibrated in lysis buffer. The column was washed with 5 column volumes (CV) lysis buffer and 5 CV wash buffer (20 mM HEPES/NaOH pH 7.5, 500 mM NaCl, 40 mM imidazole) and the protein was eluted in 5 CV elution buffer (20 mM HEPES/NaOH pH 7.5, 500 mM NaCl, 500 mM imidazole). His_6_-tagged SUMO protease was added at a 1:50 ratio (w/w) and the proteins were dialyzed overnight at 4 °C to a low imidazole buffer (20 mM HEPES/NaOH pH 7.5, 150 mM NaCl, 20 mM imidazole). The protein was subsequently applied to a re-equilibrated Ni-NTA column to remove the residual His_6_-SUMO tag and SUMO protease. The flow-through was collected, concentrated using an Amicon filter concentrator of 10 kDa cut-off (Millipore) and applied on a HiLoad™ 16/600 Superdex™ 75 preparative grade column connected to an ÄKTA Pure chromatography system (Cytiva). Protein was eluted in SEC buffer (20 mM HEPES/NaOH pH 7.5, 150 mM NaCl) at a flow-rate of 1 ml/min and 1 ml fractions were collected. Pure protein fractions were pooled, concentrated, flash-frozen in liquid nitrogen and stored at −70 °C. Point mutants of this construct were purified using the same protocol; however, for artificial cysteine variants, IMAC column buffers were complemented with 2 mM β-mercaptoethanol and the SEC buffer was complemented with 2 mM dithiothreitol (DTT) to hinder unspecific disulfide bridge formation.

Mic60 CC bacterial pellets were resuspended in lysis buffer (20 mM HEPES/NaOH pH 7.5, 500 mM NaCl, 1 mM Ethylenediaminetetraacetic acid (EDTA)), supplemented with 500 μM AEBSF and 1 μM DNase, lysed by sonication, and centrifuged as described for Mic60 HB. The filtered supernatant was applied to Dextrin Sepharose HP beads (Cytiva), washed with 5 CV of lysis buffer, and eluted using lysis buffer supplemented with 10 mM maltose. The eluate was concentrated using an Amicon filter concentrator of 30 kDa cut-off and applied to a Superose™ 6 Increase 10/300 GL column connected to an ÄKTA Pure chromatography system (Cytiva) using the above SEC buffer. The protein construct CC_1 (hsMic60 residues 473–542) eluted in an earlier shoulder peak at volume 14.2 ml and a main peak at volume 15.3 ml. The two peaks were concentrated separately as samples ‘shoulder’ (3 mg/ml) and ‘main’ (20 mg/ml). For the construct hsMic60 CC_2 (hsMic60 residues 473–617), only one peak was observed and concentrated to 35 mg/ml.

A human MBP-tagged Mic60 HB-CC construct (hsMic60 198-542) was expressed in BL21(DE3) *E. coli* as described for the CC construct. A soluble extract was applied to Dextrin Sepharose HP beads (Cytiva), which were washed with 5 CV lysis buffer. Beads were resuspended in 2 CV lysis buffer, 0.1 mg of HRV-3C protease was added and the beads were incubated under gentle rotation at 4 °C overnight. The following day, the flow-through was collected. The flow-through was concentrated using an Amicon filter concentrator of 10 kDa cut-off (Millipore) and SEC was performed as described above using a HiLoad™ 16/600 Superdex™ 200 column connected to an ÄKTA Pure chromatography system (Cytiva).

For all protein samples, purity was confirmed by mass spectrometry as described previously^38^ and samples were flash-frozen in liquid nitrogen and stored at −70 °C.

### Crystallization, data collection, refinement and structural analysis tools

Mic60 HB crystallization trials were performed using the sitting-drop vapor-diffusion method in a 96-well crystallization plate using a Gryphon pipetting robot (Matrix Technologies Co.) by mixing 200 nl protein with 200 nl reservoir solution and equilibrating against 80 μl reservoir. Commercial screens, namely JBScreen Basic HTS, JBScreen JCSG + HTS, JBScreen PEG/Salt (all Jena Bioscience), Classics II Suite, pH Clear Suite I and II, Protein Complex Suite (Qiagen), were used for initial screening at 4 °C and 20 °C using a concentration of 13 mg/ml. The Rock Imager 1000 storage system (Formulatrix) was used for storing and imaging the experiments. Initial hits were acquired for Mic60 HB (19-23 mg/ml) at 25% w/v PEG 3350, 0.1 M Tris/HCl pH 8.5 after incubation at 4 °C for one day. Optimization of PEG concentration (15.7–19.4% w/v) and pH (8.3–8.8) yielded protein crystals up to 300 μM in length, but with poor diffraction to 6-8 Å. Lowly diffracting crystals were used as seeds by mixing them with 120 μl 21.4% PEG 3350, 0.1 M Tris/HCl pH 8.9 and 30 μl Mic60 HB (19 mg/ml) and crushing by vortexing using a microseeding bead for 1 min^75^. Microseeding crystal trials against commercially available screens were set up at 4 °C using 200 nl Mic60 HB (19 mg/ml), 300 nl reservoir solution and 100 nl of a 1:10 dilution of the seeding solution in the sitting drop, equilibrated against 80 μl of reservoir solution. After 14 days, a crystal grown in 0.2 M ammonium citrate, 20% PEG 3350 was fished and flash-frozen in liquid nitrogen in the presence of 15% ethylene glycol as cryoprotectant.

Diffraction data were collected at a wavelength of 0.9184 Å and −173 °C on beamline 14.1 operated by the Helmholtz-Zentrum Berlin at the BESSY II synchrotron in Berlin Adlershof^76,77^. Sixteen hundred images were collected using 0.25 s exposure time, 426.6 mm detector distance and an oscillation increment of 0.1°. Data was automatically indexed, integrated and scaled using XDSAPP V. 3.1.9c^78^. Mic60 HB crystallized in space group P4_1_2_1_2 (92). To determine the resolution cut-off of the dataset, we used the standard settings of XDSAPP V. 3.1.9c^78^, employing the significance of half-dataset correlation CC_1/2_ in the highest resolution shell^79^. In addition, we processed the data with a more conservative resolution cut-off (see Supplementary Table 1 for details). The crystallographic phase problem was solved by molecular replacement with Phaser-MR implemented in the Phenix distribution (V. 1.20.1_4487) using two copies of AlphaFold2 predicted hsMic60 residues 198-378^41,80,81^. The Mic60 HB structure was finally solved employing Phenix.autobuild^82^, followed by manual model building in Coot V. 1.1.11 (ref. ^83^) and iterative model refinement with phenix.refine using non-crystallographic symmetry and Translation-Liberation-Screw rotation^84^. The final model contains two copies of Mic60 HB in the asymmetric unit, assembled in a dimer with non-crystallographic symmetry, with residues 210–261 and 271–377 visible for monomer 1 and 198–261 and 271–377 visible for monomer 2. Molprobity V. 4.0.2 (ref. ^85^) was used for final model validation, the resulting data statistic is provided in Supplementary Table 1. The structure was deposited and validated in the PDB database under PDB ID 9QWR.

Figures of the Mic60 HB and of the Mic60-Mic19 model were generated using PyMol (V. 2.5.5) (Schrödinger & DeLano 2020). PDBePISA was used for interface analysis of the Mic60 HB dimer^86^. The FoldSeek server was employed to perform protein structure alignments of the Mic60 HB, using the construct amino acid sequence as input^46^. For evolutionary conservation analysis of the Mic60 HB, Mic60 orthologues postulated to possess an HB were initially identified based on their AlphaFold2 predictions (from the AlphaFold Database https://alphafold.ebi.ac.uk/, as of June 2023). From these orthologues, eight representative organisms for the kingdom of animalia were identified (UniProt accession number in brackets: *Homo sapiens* (Q16891), *Mus musculus* (Q8CAQ8), *Danio rerio* (Q6PFS4), *Xenopus laevis* (A0A1L8HKP3), *Apis mellifera* (A0A7M7GMI1), *Drosophila melanogaster* (P91928), *Caenorhabditis elegans* (IMMT-1 Q22505, IMMT-2 Q9XXN2), *Hydra vulgaris* (A0A8B7DIZ7)) and their amino acid sequences were aligned using the ConSurf Web server using default parameters^87^. It was manually confirmed that the sequence segments aligned to the Mic60 HB were the ones predicted by AlphaFold2 to form the HB of each orthologue. The aligned HB sequence segments were extracted and imported to Jalview^88^ to generate a conservation score plot and a phylogenetic tree for the Mic60 HB after Huynen, et al.^45^. The ConSurf Server was then used, with the structure of the Mic60 HB dimer and the Mic60 HB sequence alignment as input, to generate structure conservation plots^87^.

### Oxidation of cysteines and non-reducing SDS electrophoresis

For the generation of fully reduced protein samples for disulfide bond analysis, the sample buffer was exchanged, and DTT concentration was increased to 10 mM (20 mM Tris/HCl pH 7.5, 150 mM NaCl, 10 mM DTT). For the oxidation reaction, protein samples were dialyzed overnight to CuSO_4_ oxidation buffer (20 mM Tris/HCl pH 7.5, 150 mM NaCl). 0.1 mM CuSO_4_ was added to the samples (5 mg/ml) and then incubated on ice for 1 min. The reaction was quenched by addition of 50 mM EDTA and residual CuSO_4_ and EDTA was removed using a PD-10 column (Cytiva). 10 μl sample (0.5 mg/ml) was added to 2.5 μl non-reducing SDS loading buffer (250 mM Tris pH 6.8, 10% SDS, 30% glycerol). Gels were stained using Coomassie Brilliant Blue and imaged using a Gel Doc™ XR+ Gel Documentation System. Protein bands were quantified by pixel count using ImageJ (V. 1.53k)^89^. For statistical analysis of three replicates, *t-*test was used, assuming normal distribution, and standard deviation was calculated.

### Analytical size-exclusion chromatography coupled to right-angle light scattering (SEC-RALS)

Analytical SEC-RALS was performed using an Agilent HPLC system equipped with an autosampler G1329B and an Omnisec Reveal (Malvern). A Superdex 75 Increase 5/150 GL column and a Superose 6 Increase 5/150 GL were used for Mic60 HB and Mic60 CC measurements accordingly. The column and RALS instrument were equilibrated overnight with SEC buffer (20 mM HEPES/NaOH pH 7.5, 150 mM NaCl). 100 μl sample (Mic60 HB 1 mg/ml, Mic60 CC 2 mg/ml) was injected using the autosampler and eluted at a flow rate of 0.2 ml/min at 20 °C. UV absorption at 280 nm, 260 nm, 210 nm and light scattering were measured and analyzed by the Agilent Offline and Malvern Omnisec software.

### Blue Native polyacrylamide gel electrophoresis (BN-PAGE)

Recombinant proteins were analyzed by BN-PAGE as described previously^38^. Briefly, 10 μg of purified protein were applied on a 4–16% acrylamide gradient BN-PAGE gel, which was run at 150 V for 2 h on ice and subsequently stained using Coomassie Brilliant Blue.

### In vitro cross-linking MS

Cross-linking was performed according to previously reported in vitro protocols^90^. Briefly, Mic60 HB-CC (*n* = 1) was diluted to 250 μl of 1 mg/ml using SEC buffer (20 mM HEPES/NaOH pH 7.5, 150 mM NaCl). PhoX (Thermo-Fisher-Scientific) was freshly dissolved in anhydrous DMSO to a concentration of 50 mM and added to the sample in two steps, followed by 20 min incubation on ice, reaching a final concentration of 1.2 mM. The reaction was quenched with 20 mM Tris/HCl pH 8.0 for 30 min at room temperature. After quenching, proteins were directly subjected to tryptic digestion in-solution. 8 M urea, 5 mM TCEP, and 40 mM chloroacetamide were added and the mixture was incubated at 37 °C for 1.5 h in the dark. Proteolytic digestion was performed using LysC (1:200 ratio (w/w)) and trypsin (1:100 ratio (w/w)). Prior to trypsin addition, samples were pre-digested with LysC at 37 °C for 2–4 h. The formic acid solution was then diluted to a final concentration of 2 M urea using 50 mM triethylammonium bicarbonate buffer (pH 8.5). The digestion was stopped with 1% formic acid, and peptides were desalted using C8 StageTips. Peptides were dried and stored at −20 °C until measurement.

Peptides were resuspended in 1% acetonitrile with 0.05% trifluoroacetic acid and 1 µg was injected into the LC-MS system. Separation was performed via reverse-phase (RP)-HPLC on a Thermo Scientific Vanquish neo system using a PepMap C-18 trap-column (0.075 mm×50 mm, 3 μm particle size, 100 Å pore size, Thermo Fisher Scientific) and an in-house packed C18 column (Poroshell 120 EC-C18, 2.7 μm, Agilent Technologies) at a flow rate of 250 nL/min. RP separation was achieved using a 180-min method. Samples were analyzed on an Orbitrap Exploris 480 mass spectrometer (equipped with a FAIMS Pro Duo interface (Thermo Scientific)). MS1 scans were recorded in the Orbitrap at a resolution of 120,000, 375–1400 m/z scan range, 50 ms maximum injection time, and 300% AGC target. Precursors with charge states 3 – 8 were isolated in 2 s per FAIMS CV (−50/−60/−70) cycles with a 2 m/z window and subjected to MS2 before being dynamically excluded from fragmentation for 60 s. Fragment ions were generated with NCE 30% and detected in the Orbitrap at a resolution of 30,000, automatic scan range, 100 ms maximum injection time, and 200% AGC target.

RAW files were analyzed using XlinkX^91^ implemented in Proteome Discoverer version 3.2, operating in non-cleavable mode. After recalibration of RAW files, the search parameters were set as follows: a mass tolerance of 10 ppm for MS1 and 20 ppm for MS2, with a maximum of 3 missed cleavages and a minimum peptide length of 5 with peptide masses ranging from 300 to 7000 Da. Cross-linker modifications were defined for lysine residues and protein N-termini ( + 209.97 Da). Oxidation of methionine ( + 15.995 Da) was considered as a variable and carbamidomethylation of cysteine ( + 57.021 Da) as a static modification. Searches were conducted against a database containing the target protein sequences. The false discovery rate was controlled at 1% on cross-link spectrum match (CSM)- and cross-link level, with separate estimation for intra- and inter-links. Cross-links were exported and dynamic score thresholds were applied based on the maximum decoy CSM scores, and identifications were further filtered post-search by score or minimum CSM count. All cross-links were visualized using ChimeraX with the XMAS tool^92,93^.

### Assembly of the initial Mic60-Mic19 model

A model of the Mic60-Mic19 complex was assembled to study it by molecular dynamics simulations. Three solved structures were used as the basis for this model: the Mic60 HB (residues 198–378, described in this paper), the CC domain of *Lachancea thermotolerans* Mic60 (PDB ID: 7PUZ, residues 207-382, equivalent residues in hsMic60 are residues 443–603) and a fusion construct of the Mic60 mitofilin domain with the Mic19 CHCH domain from the organism *Chaetomium thermophilum* (PDB ID: 7PV1, ctMic60 565-586-GS-622-691 and ctMic19 residues 116-164, equivalent residues are hsMic60 residues 627–751 and hsMic19 residues 180–222).

To utilize the previously published fungal orthologue structures in assembling the human Mic60 model, homologous structures were generated using SWISS-MODEL, covering the residue range included in the experimental models^48^. According to the AlphaFold2 database (https://alphafold.ebi.ac.uk/entry/AF-Q16891-F1, Q16891, generated on 18th of November 2022)^41^, the remaining sequence segments were predicted to be the N-terminal intrinsically disordered region (residues 1–197, including the transmembrane helix), an extended helical connection between the Mic60 HB and the CC (residues 379–442), short unfolded linker regions and the flexible LBS2 (residues 649–685). The majority of these segments, apart from the helical connection, was predicted to be unstructured and included in the final model according to the AlphaFold2 prediction of full-length hsMic60.

Structural information for hsMic19 is only available for the CHCH domain, leading us to exclude the remaining protein sequence in the final Mic60-Mic19 model to avoid inaccurate model building. The SWISS-MODEL generated homolog was modeled on the mitofilin domain of the hsMic60 model according to the previously published structure on their interaction (PDB ID: 7PV1).

The SWISS-MODEL orthologues and AlphaFold2 predictions were manually integrated using Coot and spatially adjusted using iterative cycles of ChimeraX V. 1.9 (ref. ^93^), ultimately assembling a model of the human Mic60-Mic19 hetero-octamer. During this process, particular attention was paid to ensure that the structural elements retain their experimentally determined characteristics, e.g., their oligomerization state. Some short structural segments between the CC and the LBS1 (residues 527–536, 555–561, 584–591 and 603–607) predicted by AlphaFold2 to be helical were reconstructed as flexible linkers in the molecular dynamics simulations.

While the model reflects the known structural information on individual domains of hsMic60, there is no experimental data on the relative positioning of these domains to one another. The oligomeric state of the individual domains results in the CC forming a tetrameric core, as illustrated previously for fungal Mic60 (ref. ^38^). The short linker length to the LBS-mitofilin domain as well as the directionality of the LBS domains pointing away from the complex and towards the hypothetical inner membrane lead to a planar orientation for these domains of the tetramer. Neither experimentally nor computationally could it be determined how the hsMic60 CC tetramer is connected to the hsMic60 HB dimers and how the HB dimers are positioned relative to the tetrameric CC. Therefore, two models were generated. In model 1, the HB domains and mitofilin domains dimerized via different Mic60 molecules, whereas in model 2, the same molecules mediate dimerization of the two domains (Supplementary Fig. 4c, d). Graphical representations of the two models were prepared using Affinity Designer 2 (V. 2.6.5).

### Molecular dynamics simulations of the Mic60-Mic19 model

For all simulations, the N-terminal region of Mic60 exposed to the matrix as well as the transmembrane domain, corresponding to the first 61 residues, was removed, making Ile62 the N-terminus of cristae-soluble Mic60. To fill the sequence gaps and the overstretched regions, a homology modeling process was performed using Modeler^94^ and a 4-fold structural symmetry was subsequently applied to the initial model, using a custom protocol to apply symmetry restraints to the Cα atoms. The Modeler MD refinement protocol was performed at the *slow* level. A total of 300 models were generated, picking the one with the lowest DOPE score^95^.

All-atoms molecular dynamics simulations of the Mic60-Mic19 complex were set up using OpenMM^96^. The system was aligned along its principal axis and immersed in solvent within a simulation box measuring 27.5 × 19.8 × 15.3 nm^3^. The solvent consists of TIP3P water molecules neutralized with Na^+^ and Cl^-^ ions at 0.15 M ionic strength^97^. Non-bonded interactions are treated with Particle Mesh Ewald, cut-off of 1 nm and a 0.75 nm switching distance. Hydrogen bonds are constrained, and hydrogen mass was set to 4 atomic mass units (amu) to enable larger simulation timesteps. The force field employed is the Amber14SB force field^98^. The system was slowly equilibrated using a multi-step protocol to ensure stability under physiological conditions. The system was subjected to energy minimization before gradual thermal and pressure equilibration with Langevin Middle Integrator and a 2 fs timestep. Harmonic positional restraints of 500 kJ/mol/nm² were initially applied to the protein heavy atoms. Equilibration began with an NVT warm-up phase (NVT for number (N), volume (V), and temperature (T)) lasting 200 ps, during which the temperature was gradually increased from 10 K to 300 K over 100,000 steps. The system then underwent NPT equilibration for 400 ps with a Monte Carlo barostat set at 1 bar and 300 K, maintaining the initial restraints throughout 200,000 steps. To progressively relax constraints, the harmonic restraint force was then reduced from 500 kJ/mol/nm² to 100 kJ/mol/nm² over 400 ps gradually. Positional restraints were removed from side chains and applied exclusively to the backbone, maintaining a force constant of 100 kJ/mol/nm² for 400 ps. Finally, the backbone restraints were gradually reduced to zero over 600 ps. Positional restraints were removed from side chains and applied exclusively to the backbone, maintaining a force constant of 100 kJ/mol/nm² for 400 ps. Finally, the backbone restraints were gradually reduced to zero over 600 ps.

Starting from the equilibrated structure, 30 simulations were run to maximize the exploration of the conformational space, increasing the timestep to 4 fs and saving frames every 0.04 ns. Simulations were run for around 500 ns until RMSD equilibration with respect to the common initial structure, as shown in Fig. 2c.

For the alternative model, the same procedure described above was applied. The system was solvated in a 36.6 × 27.6 × 20.2 nm simulation box, larger than that used for the main model, to accommodate its increased longitudinal dimension. Five independent simulations were subsequently performed, each extending for approximately 170 ns.

Using OpenMM’s CustomExternalForce, each of the 30 final structures was gradually coupled to an external potential that emulates a crista junction, modeled as a concatenation of a cylindrical and catenoid geometry^96^. The CJ was defined with a diameter of 17 nm. Attachment of Mic60 was achieved at the N-terminus by linking the Cα atom of the first residue (Ile62) to the potential. For the C-terminus, two attachment points per chain were selected, corresponding to the Cα atoms of residues flanking the LBS2 region (Arg652, Pro670). These points were tethered via a harmonic potential with a force constant of 200 kJ/mol/nm² applied perpendicular to the crista surface, allowing free tangential mobility along the crista. The coupling was implemented incrementally, with the force constant ramped from 0.1 kJ/mol/nm² to 200 kJ/mol/nm² over ten steps of 5 ns each. Following the equilibration phase of the attachments, a production simulation of 100 ns was conducted. The 30 all-atom trajectories, after attachment to catenoid and equilibration, were used to study the contacts formed between the Mic60 HB and CC domains, as they were the last simulation time-points which resolved side-chain conformations. We defined two residues to be in contact if their non-hydrogen atoms were closer than 0.45 nm (ref. ^99^) and computed the residues in contact across the 30 final frames and for the 16 combinations of the HB and CC (Supplementary Fig. 7e). The most consistent interface was that between the HB aa 278–311 and the CC aa 416–456, with a surface area of 3075 Å^2^ as calculated with PyMol (V. 3.0, Schrödinger, LLC. 2025). hsMic60 conservation was calculated for the full-length protein using ConSurf with standard settings across all known Mic60 orthologues and visualized on the interface using PyMol (V. 3.0, Schrödinger, LLC. 2025)^87^ (Supplementary Fig. 7f).

MLCG simulations were performed using the chemically transferable model published in ref. ^51^. This model employs a coarse-grained representation with a resolution of five beads per residue. All information required to run simulations with the *mlcg* package is publicly available in the Code Availability section of ref. ^51^. For the Mic60-Mic19 complex, five independent simulations were conducted, each consisting of 2,000,000 timesteps. Simulations were carried out using a Langevin integrator at a temperature of 300 K, with a friction coefficient of 1.0 and a timestep of 4 fs. Positions were saved every 10 timesteps. Further information on the number of atoms in each system setup of the simulations is found in Supplementary Table 2.

### In silico force calculation of Mic60-Mic19 binding to the IMM

To evaluate the interaction strength of the complex with the IMM, we conducted coarse-grained molecular dynamics simulations using the Martini force field^100^ and performed umbrella sampling to quantify the free energy of binding. We focused on the Mic60-Mic19 C-terminal mitofilin-CHCH lobe, which was prepared using the martinize2 script with the Martini 3 force field, incorporating a Go-like network to preserve secondary structure and native contacts^101^. The system was solvated using the insane script^102^, with a simulation box size of 17 × 23 × 35 nm³ and a lipid composition of POPC:POPE:CDL0:POPS in a 40:40:15:5 ratio to mimic the IMM^103^. Na⁺ and Cl⁻ ions were added to achieve an ionic concentration of 0.15 M. The system was then simulated for 1 µs using martini_openmm^104^ at 300 K, with a friction coefficient of 10 ps⁻¹, a timestep of 20 fs, a nonbonded interaction cut-off of 1.1 nm, and a barostat set to 1 bar. To simulate the curvature of the crista membrane, we first removed the solvent and applied membrane bending using the expression described in ref. ^105^, with a γ factor of 0.05. The system was then re-solvated to accommodate the new membrane dimensions. To preserve the membrane curvature, the barostat was set to 1 bar, acting exclusively along the z-dimension. Additionally, an OpenMM CustomExternalForce was implemented to apply a harmonic potential with a force constant of 200.0 kJ·mol⁻¹·nm⁻² on the headgroups of the lower leaflet. The system was subsequently equilibrated for 1000 ns using the same simulation parameters as described above.

The initial window frames for umbrella sampling were generated using steered molecular dynamics. The protein was pulled 10 nm away from the membrane using a CustomCentroidBondForce between the center of mass (COM) of the protein and the membrane, with a force constant of 1000.0 kJ·mol⁻¹·nm⁻² and a pulling speed of 1 × 10⁻⁵ nm/ps. To constrain lateral motion, a harmonic restraint was applied along the x and y directions to the COM of the protein, with a force constant of 200.0 kJ·mol⁻¹·nm⁻². Initial configurations along the reaction coordinate were extracted by defining umbrella sampling windows separated by 0.1 nm. Starting from the equilibrated position, five windows were placed in the direction of the protein closer to the membrane, while the remaining 50 were positioned progressively further away. Each window was then simulated for 1000 ns. The COM distance time series was analyzed using the weighted histogram analysis method (WHAM) (Grossfield, Alan, “WHAM: the weighted histogram analysis method”, V. 2.1.0, http://membrane.urmc.rochester.edu/wordpress/?page_id=126). As shown in Supplementary Fig. 7a, the free energy difference upon binding is approximately 100 kJ/mol, which was used to parameterize the interaction strength of the attachment points in the crista potential described above.

### *In organello* cross-linking as validation for the Mic60-Mic19 model

Cross-linking mass spectrometry data from Zhu, et al.^56^ (accessible through PRIDE accession number PXD046382, see Source data of Fig. 3a for an extensive overview of cross-link pairs, CSM scores and n_scores) were employed to validate the ensemble of generated structures. To speed up conformational explorations, Cα structure-based simulations were started from the 30 all-atom final structures^55^. The standard Cα structure-based angle potential was replaced with a restricted angle potential for stability^106^. Native contacts were computed for each starting configuration using the Shadow algorithm^107^. Native contacts formed involving the IDR for all the chains were excluded. Simulations were run for 100 ns with a timestep of 0.5 fs and a reduced temperature of 0.5 (ref. ^108^). The cross-link satisfaction cut-off was set to 40 Å to account for cross-linker length, lysine side-chain flexibility and backbone dynamics^56^.

Due to the multimeric assembly of the Mic60-Mic19 complex, there were 16 possibilities for residue-residue cross-links and six possibilities for self-cross-links. We used ResPairs and “Find shortest” in ChimeraX to identify the most proximal possible cross-linked subunit. To account for the multiplicity of chains, we considered all possible crosslinks and plotted their distance distribution across simulation frames as violin plots in Supplementary Fig. 8. The cross-link satisfaction percentage was calculated two-fold. Firstly, for individual experimental set-ups, it was calculated cumulatively across all simulation frames and considered satisfied when two residues of any chain fulfilled the distance cut-off for at least 1% of frames at any time up to the indicated time point. This provided better insight into the overall proximity of the lysine pairs throughout the simulations. Secondly, for comparing different set-ups, such as the main model with the alternative model or with the MLCG simulations, cross-link satisfaction was calculated, at a given time, in consideration of the mean and standard deviation of the lysine distance over the independent runs, to account for the differences in the number of independent trajectories per experimental set-up.

Cross-links were plotted on the hsMic60 sequence using xiNET^109^. The cross-link identifications were converted into a ChimeraX XMAS-compatible output^92^ and then visualized on protein models, using the option “Find shortest” in ChimeraX to only show the most proximal cross-link for ease of illustration.

Zhu, et al.^56^ provide a further mitochondrial cross-linking dataset using the common cross-linker disuccinimidyl sulfoxide (DSSO) (accessible through PRIDE accession number PXD032132, see also Source data for a detailed list of residue-residue pairs). This dataset was analyzed as described above for DSBSO, using a cut-off of 35 Å, and the lysine-lysine cross-link matches between the two datasets were compared.

The cross-link satisfaction of the alternative model was lower than that of the main model (Supplementary Fig. 5e), leading us to prioritize the main model in subsequent analyses.

### In silico CJ diffusion experiments

The trajectory that satisfied the highest number of cross-linkers was selected for diffusion-like simulations. In the Cα structure-based system, 200 identical spheres, each with the same radius, were introduced above the Mic60-Mic19 plane. A harmonic potential was applied to confine the spheres within the crista junction potential. To impose additional constraints, upper and lower planes were set to restrict the spheres within a closed space. No interaction was set between the spheres, while a Lennard-Jones repulsion term, proportional to $\frac{{r}_{{sphere}}}{{r}^{12}}$, was introduced with Mic60-Mic19 and with the crista potential. The crossing event frequency was defined as the fraction of frames exhibiting an above-to-below Mic60-plane transition at equilibrium, normalized by the total number of frames, and was studied in correlation to the sphere radius. To approximately correlate particle radius to protein molecular weight, we used the spherical approximation with a density of 1.35 g/cm^3^ (ref. ^59^).

### Statistics and reproducibility

No statistical method was used to predetermine sample size, instead, they were decided according to literature standards. We performed three replicates for biophysical and biochemical experiments (non-reducing SDS, SEC-RALS, BN-PAGE), 30 parallel simulations for the molecular dynamics simulations of the main model to ensure a detailed exploration of its conformational variability (in all-atom and Cα-based simulations), and five parallel simulations for the MLCG simulations of the main model as well as five parallel simulations of the all-atom simulations of the alternative model to be able to compare them to the main model with statistical confidence. For the diffusion experiments, a similar strategy was followed, with a sample size of 200 spheres. No data were excluded from the analyses. The experiments were not randomized and the investigators were not blinded to allocation during experiments and outcome assessment.

## Supplementary information

Supplementary Information

Transparent Peer Review file

Description of Additional Supplementary Files

Supplementary Video 1

## Source data

Source data

## Data Availability

The atomic coordinates of Mic60 HB were deposited in the Protein Data Bank with accession number 9QWR. The atomic coordinates of previously resolved Mic60 structures are deposited in the Protein Data Bank with accession numbers 7PUZ and 7PV1, and the Smac/DIABLO structure used for alignment in Supplementary Fig. 3d is 1FEW. For the molecular dynamics simulations, initial coordinate as well as simulation input and output files are provided in the public repository Zenodo [https://doi.org/10.5281/zenodo.21871799]. The *in organello* cross-linking datasets re-evaluated during this study are published in Zhu, et al.^56^ and can be accessed through PRIDE accession number PXD046382 (DSBSO) [https://www.ebi.ac.uk/pride/archive/projects/PXD046382] and PXD032132 (DSSO) [https://www.ebi.ac.uk/pride/archive/projects/PXD032132]. The in vitro cross-linking mass spectrometry proteomics data have been deposited to the ProteomeXchange Consortium via the PRIDE partner repository^110^ with the dataset identifier PXD077526. Source data underlying Figs. 1c, 1d, 2b, 2c, 3a, 3b and 4b, 4c, as well as Supplementary Figs. 2-10 are provided with this paper as a Source data file. Source data are provided with this paper.
