# Supplementary material for: Integrative structural modelling reveals the human Mic60-Mic19 subcomplex as a diffusion barrier in mitochondria
Source: Nat Commun. 2026 Sep 26;17:10234. doi: 10.1038/s41467-026-77869-3 (PMC13615979; doi:10.1038/s41467-026-77869-3)
Supplement: Supplementary file 1 [file 41467_2026_77869_MOESM1_ESM.pdf]

Supplementary Information for

Integrative structural modelling reveals the human Mic60-Mic19 subcomplex as a diffusion barrier in mitochondria

Evangelia Nathanail, Edoardo Rolando, Max Ruwolt, Iryna Zaporozhets, Fan Liu, Cecilia Clementi, Oliver Daumke

This PDF file includes:

Supplementary Figures 1 to 10

Supplementary Table 1 for crystallographic data collection and refinement statistics

Supplementary Table 2 for molecular dynamics system setups

References of the Supplementary Information

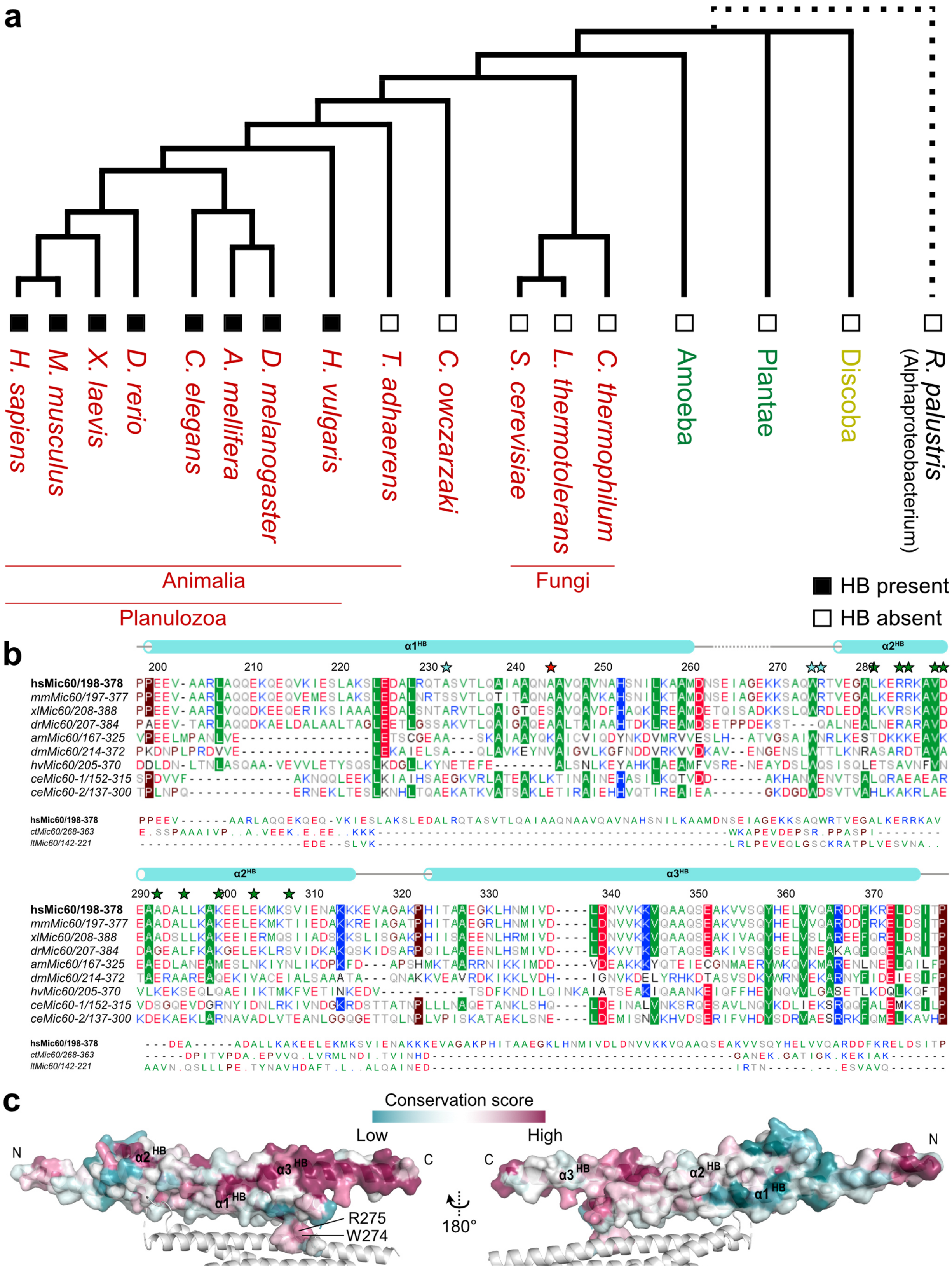

Supplementary Figure 1

Supplementary Figure 1: Evolutionary conservation analysis of the N-terminal helical bundle

a Phylogenetic tree depicting the taxonomical relationship among the examined organisms (UniProt accession number in brackets): *Homo sapiens* (Q16891), *Mus musculus* (Q8CAQ8), *Xenopus laevis* (A0A1L8HKP3), *Danio rerio* (Q6PFS4), *Caenorhabditis elegans* (IMMT-1 Q22505, IMMT-2 Q9XXN2), *Apis mellifera* (A0A7M7GMI1), *Drosophila melanogaster* (P91928), *Hydra vulgaris* (A0A8B7DIZ7), *Trichoplax adhaerens* (B3S3B5), *Capsaspora owczarzaki* (A0A0D2UAX3), *Saccharomyces cerevisiae* (P36112), *Lachancea thermotolerans* (C5E325), *Chaetomium thermophilum* (G0SHY5). Dotted line shows unsure rooting of mitochondria with alphaproteobacteria. Organisms that possess the HB are marked with a black square, organisms expressing Mic60 but not predicted to possess a HB are marked with an empty square. **b** Sequence conservation analysis of the hsMic60 HB shows high sequence similarity among vertebrates (*Homo sapiens (hs)*, *Mus musculus (mm)*, *Danio rerio (dr), Xenopus laevis (xl)*) and partial sequence preservation in protostomia (*Apis mellifera (am), Drosophila melanogaster (dm)*, *Caenorhabditis elegans (ce)*, *Hydra vulgaris (hv)*). Highly conserved residues (> 60%, calculated based on orthologues where the HB is present) are placed in coloured boxes, individual residues are coloured according to their physicochemical properties (hydrophobic in green, positively charged in blue, negatively charged in red, prolines and glycines in brown, remaining in grey). Residues mutated in the current study are indicated by cyan stars, artificial cysteine bridge indicated by a red star, residues shown to contact the CC domain in MD simulations indicated by green stars. A sequence alignment of hsMic60 with fungal orthologues below illustrates that they lack a HB and show low sequence similarity to the equivalent human sequence, shown here for *Chaetomium thermophilum* (*ct*) and *Lachancea thermotolerans* (*lt*) Mic60. **c** Surface representation of the Mic60 HB coloured by the ConSurf conservation score (cyan-to-purple colouring variable-to-conserved). The W274-containing loop and a patch of $\alpha3^{HB}$ comprise highly conserved surfaces in the HB, pointing to their functional relevance.

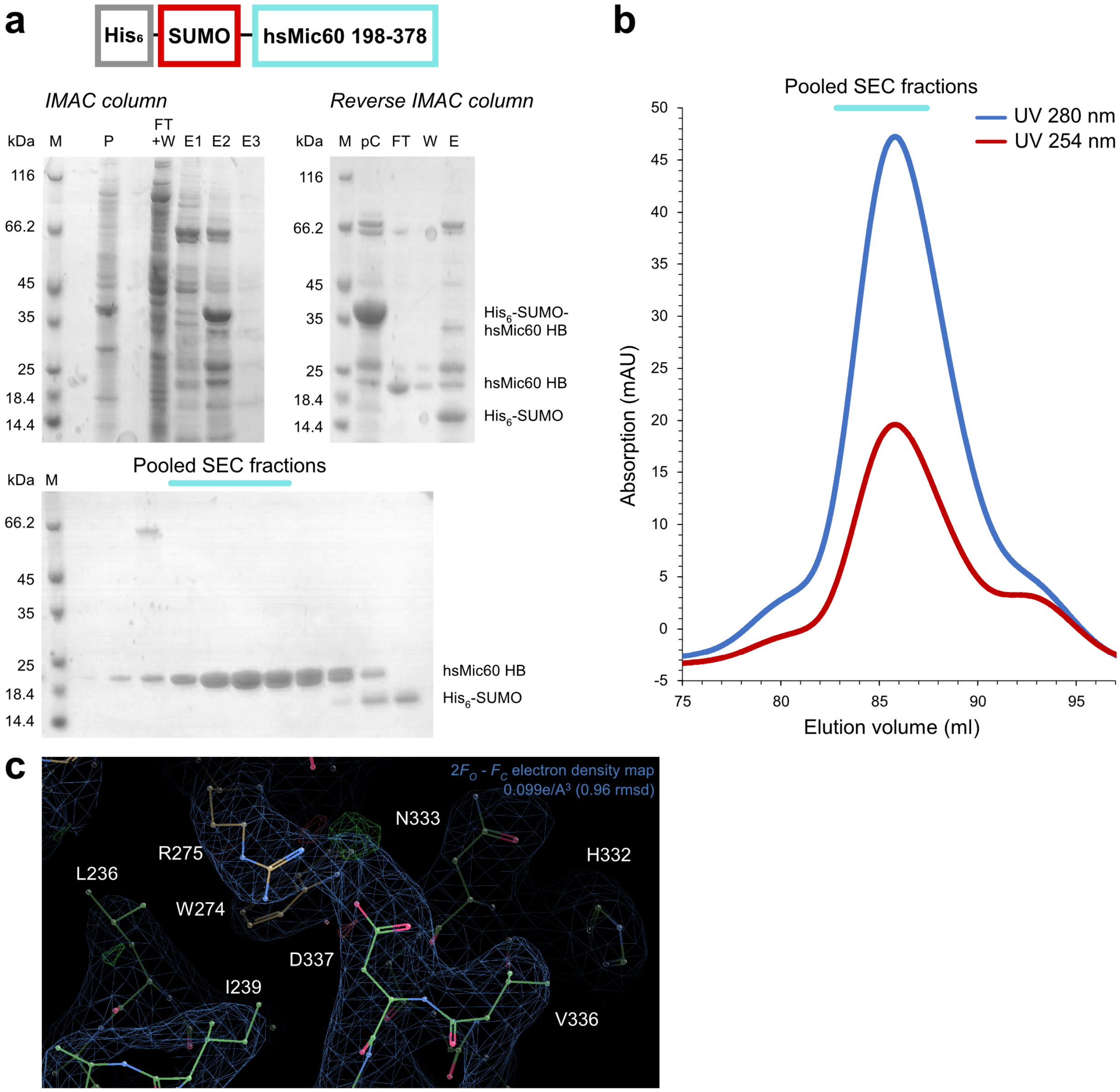

Supplementary Figure 2: Purification of the Mic60 helical bundle (Mic60 HB)

a Schematic overview of the Mic60 HB with an N-terminal His_6-SUMO tag. The protein was purified by Immobilized Metal Affinity Chromatography (IMAC, left SDS gel), followed by overnight cleavage using SUMO protease and a further IMAC (right SDS gel). Descriptions on the left side of the gel indicate the molecular weight of the marker (M) proteins, descriptions on the right side indicate the deduced position of the cleaved and non-cleaved target proteins and the His_6-SUMO tag. The final pure sample was acquired after SEC elution (bottom SDS gel). The cyan bar indicates the pooled fractions. P – Pellet, FT – Flow through, W – Wash, E – Eluate, pC – pre-cleavage. **b** Chromatogram of the SEC elution profile (HiLoad™ 16/600 Superdex™ 75) of Mic60 HB. Absorption at 280 nm (dark blue) and 254 nm (red) are plotted over the peak elution volume. The cyan bar indicates the pooled fractions shown in a. **c** Image of a portion of a $2F_o$-F_c electron density map of the Mic60 HB structure, showing the insertion loop.

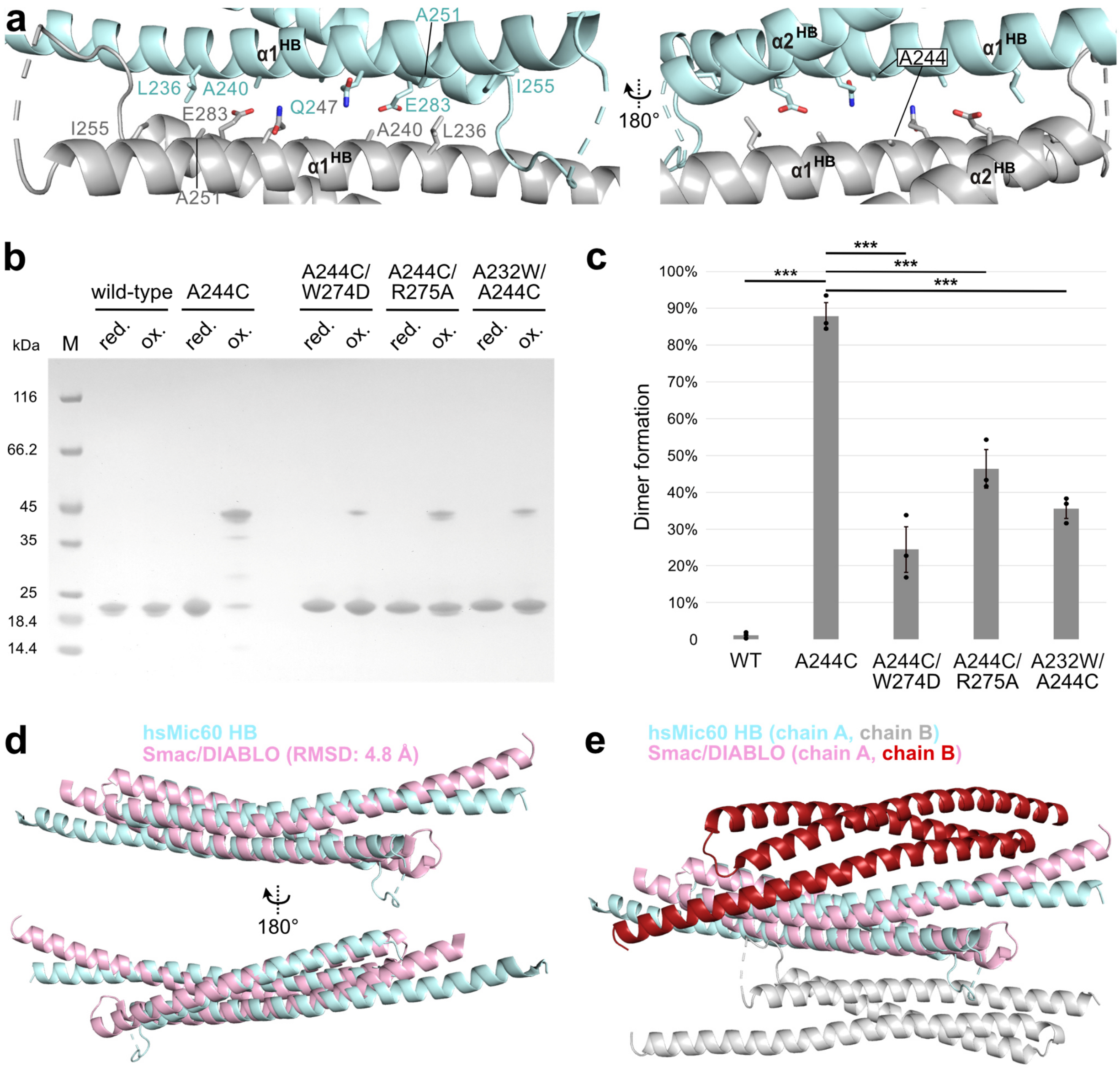

Supplementary Figure 3: Mic60 HB dimerization is captured by the artificial cysteine bridge A244C and differs from structurally homologous Smac/DIABLO

a Close-up view of the dimerization interface of Mic60 HB. It centres around an antiparallel hydrophobic patch of $\alpha 1^{HB}$ involving residue A244, which was used for designing artificial cysteine variants of the Mic60 HB. **b** Independent replicate experiment of the experiment shown in Fig. 1d: Non-reducing SDS PAGE of Mic60 HB cysteine mutants before and after $CuSO_4$ oxidation (from left to right: wt, A244C, A244C/W274D, A244C/R275A, A232W/A244C). Lower bands correspond to a monomer, upper bands correspond to a dimer formed through the artificial cysteine bridge. **c** Dimer formation quantification after $CuSO_4$ oxidation for Mic60 HB wild-type and cysteine mutants using non-reducing SDS PAGE (see Fig. 1d). Bars indicate the standard deviation from triplicate gels, with individual data points shown as circles. *** $p < 0.001$, calculated by t-test from three replicates. **d** Smac/DIABLO (PDB ID: 1FEW [https://doi.org/10.2210/pdb1FEW/pdb]) was identified as a homologous structure to the Mic60 HB using Foldseek[1] (RMSD = 4.8 Å, Smac/DIABLO monomer coloured pink). **e** Mic60 HB and Smac/DIABLO deploy different dimerization interfaces (Mic60 HB dimer coloured in cyan and grey, Smac/DIABLO dimer coloured in pink and red).

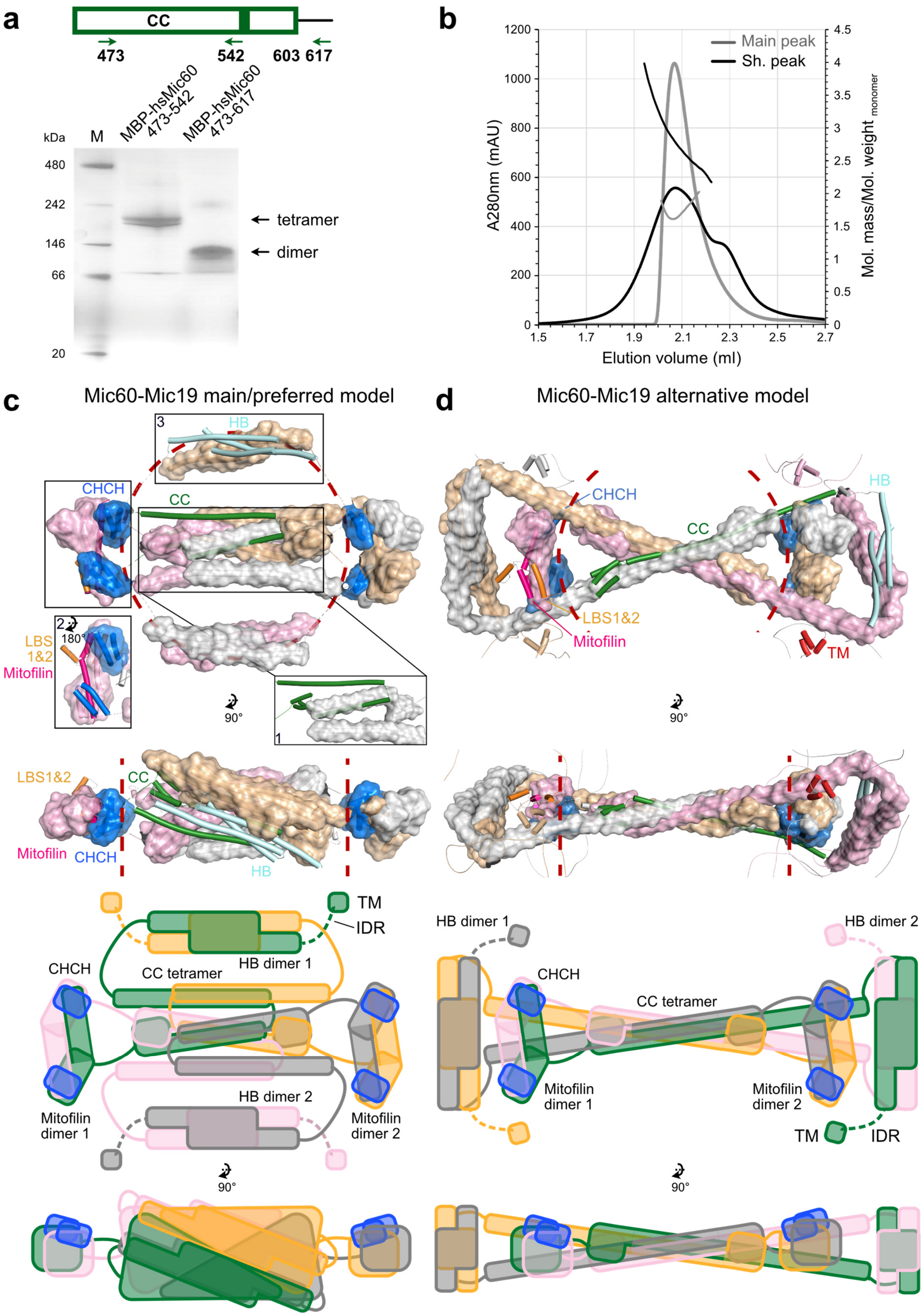

Supplementary Figure 4

Supplementary Figure 4: hsMic60 can form tetramers similarly to *Lachancea thermotolerans* Mic60, leading to two possible Mic60-Mic19 complex assemblies

a Graphic illustration of the two hsMic60 CC constructs used in this study: CC_1 hsMic60 473-542 and CC_2 hsMic60 473-617. BN-PAGE analysis shows the truncated CC_1 construct can form tetramers while CC_2 can form only dimers. Experiment performed in triplicate. **b** SEC-RALS profile of CC_1. The protein was separated during purification into an earlier shoulder peak (grey) and a main peak (black) and the peaks were pooled separately. They were analysed again via SEC-RALS and eluted in a dimer-tetramer equilibrium and as a dimer, respectively. **c** The main homology-based model of the human Mic60-Mic19 complex (above) along with a graphical cartoon (below), as shown in Fig. 2a. **d** An alternative conformation of the model of the human Mic60-Mic19 complex. The model is coloured as in **c** and fulfils the same structural restraints as the first model. Here, the two dimeric HB modules are assembled by the same Mic60 moieties that dimerize through their mitofilin domains, resulting in a more extended conformation.

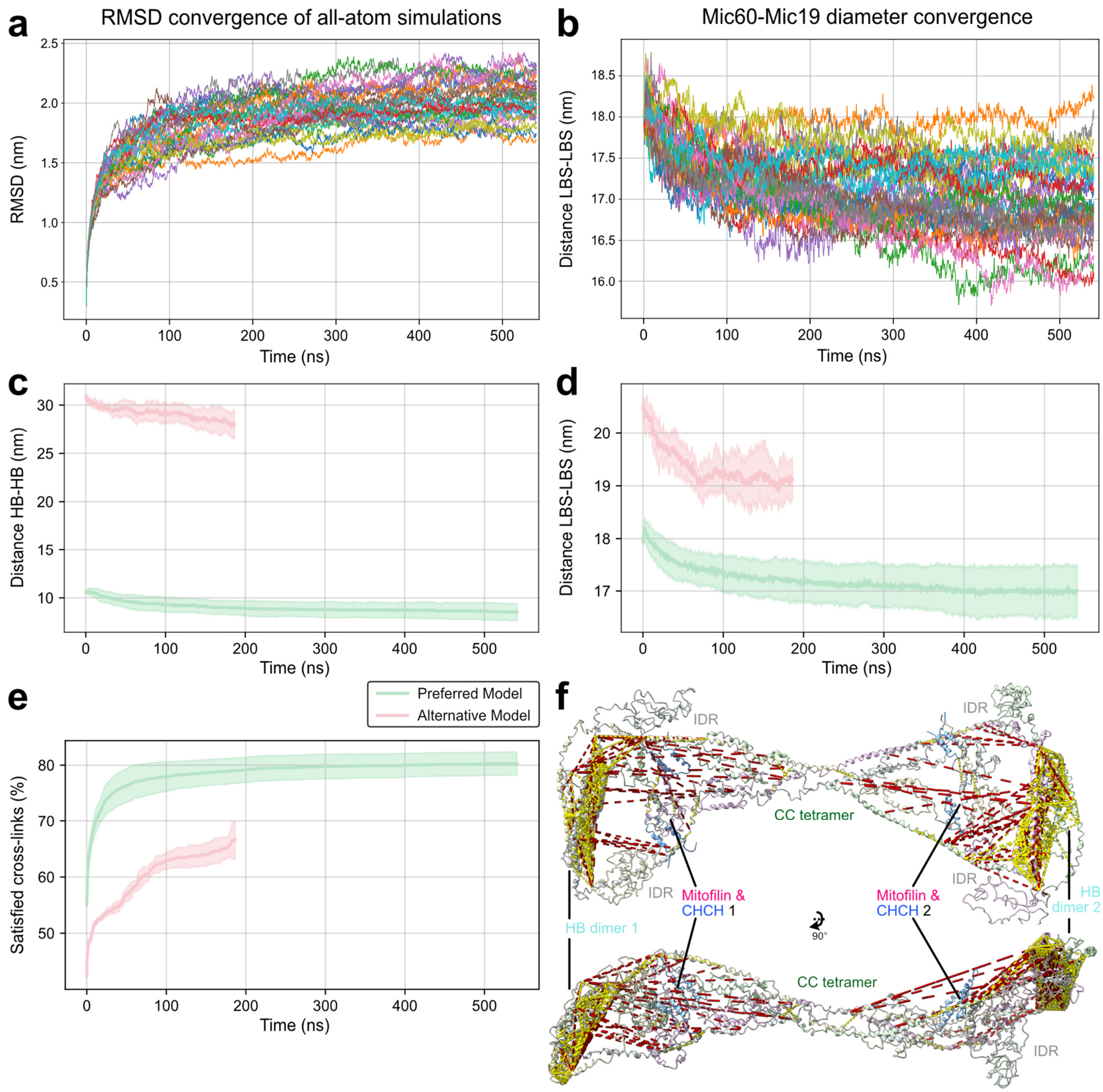

Supplementary Figure 5: Equilibration graphs (RMSD, HB-HB and LBS-LBS distance) and cross-link validation favour the main model of the human Mic60-Mic19 complex over the alternative model
a RMSD of each of the 30 all-atom simulations is shown over the time course of the simulation, resulting in the average and standard deviation of Fig. 2b. **b** LBS-LBS distance for each of the 30 all-atom simulations, resulting in the average and standard deviation of Fig. 2c. **c, d** Diameter convergence of the main and alternative human Mic60-Mic19 complex models during the all-atom simulations, prior to the attachment to the catenoid, plotted for the HB-HB distance (**c**) and the LBS-LBS distance (**d**). Line represents the mean and shading represents the standard deviation across five independent trajectories. **e** The percentage of cross-links from the DSBSO cross-linking dataset satisfied across the frames of the all-atom simulation of the main and the alternative models, considering, at a given time, the mean and standard deviation over the independent runs for all possible cross-links (line representing the mean and shading representing the standard deviation across five independent trajectories for the alternative model and thirty independent trajectories for the main model). **f** The DSBSO cross-linking dataset (excluding IDR cross-links for ease of illustration) plotted on the alternative homology-based Mic60-Mic19 model after the all-atom simulations (yellow for in-range, red for out-of-range, cut-off set to 40 Å).

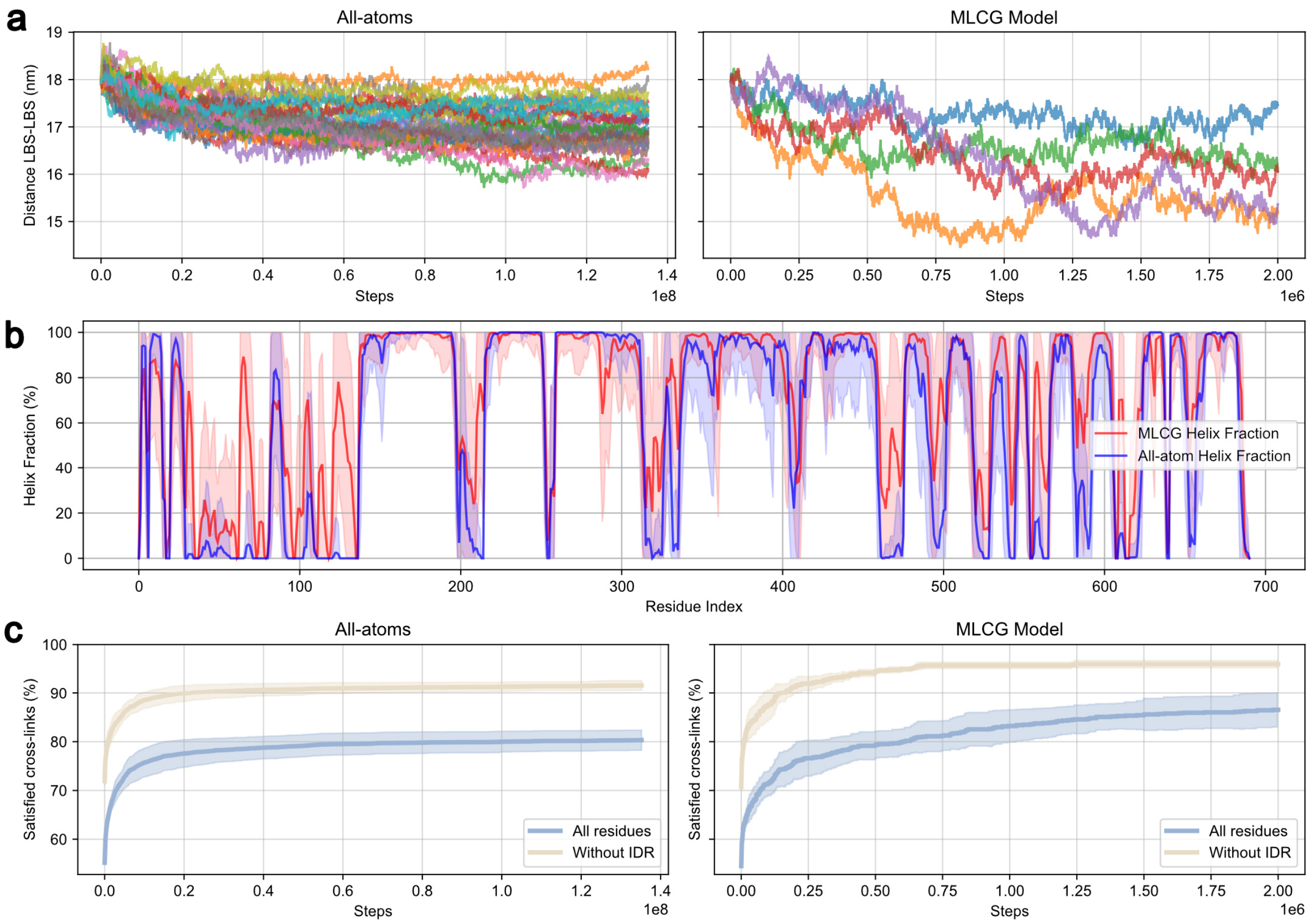

Supplementary Figure 6: Machine-learned coarse-grained (MLCG) model simulations of the main model
a Diameter convergence (plotted as LBS–LBS distance) across the steps of the simulation, comparing the all-atom (also shown in Supplementary Fig. 5b) and MLCG simulations. The MLCG (right) simulations exhibit a more pronounced structural compression and greater variability across independent trajectories compared to the all-atom model (left). **b** Helical content per residue, determined using coarse-grained DSSP in MDTraj[2] and shown as the mean and standard deviation over the simulation, highlights the correspondence between the all-atom (blue) and MLCG (red) models. **c** The percentage of DSBSO cross-links satisfied across the steps of the all-atom or MLCG simulations, considering, at a given time, the mean and standard deviation over the independent runs for all possible cross-links. Line represents the mean and shading the standard deviation across thirty independent trajectories for the all-atom simulations and five independent simulations for the MLCG model.

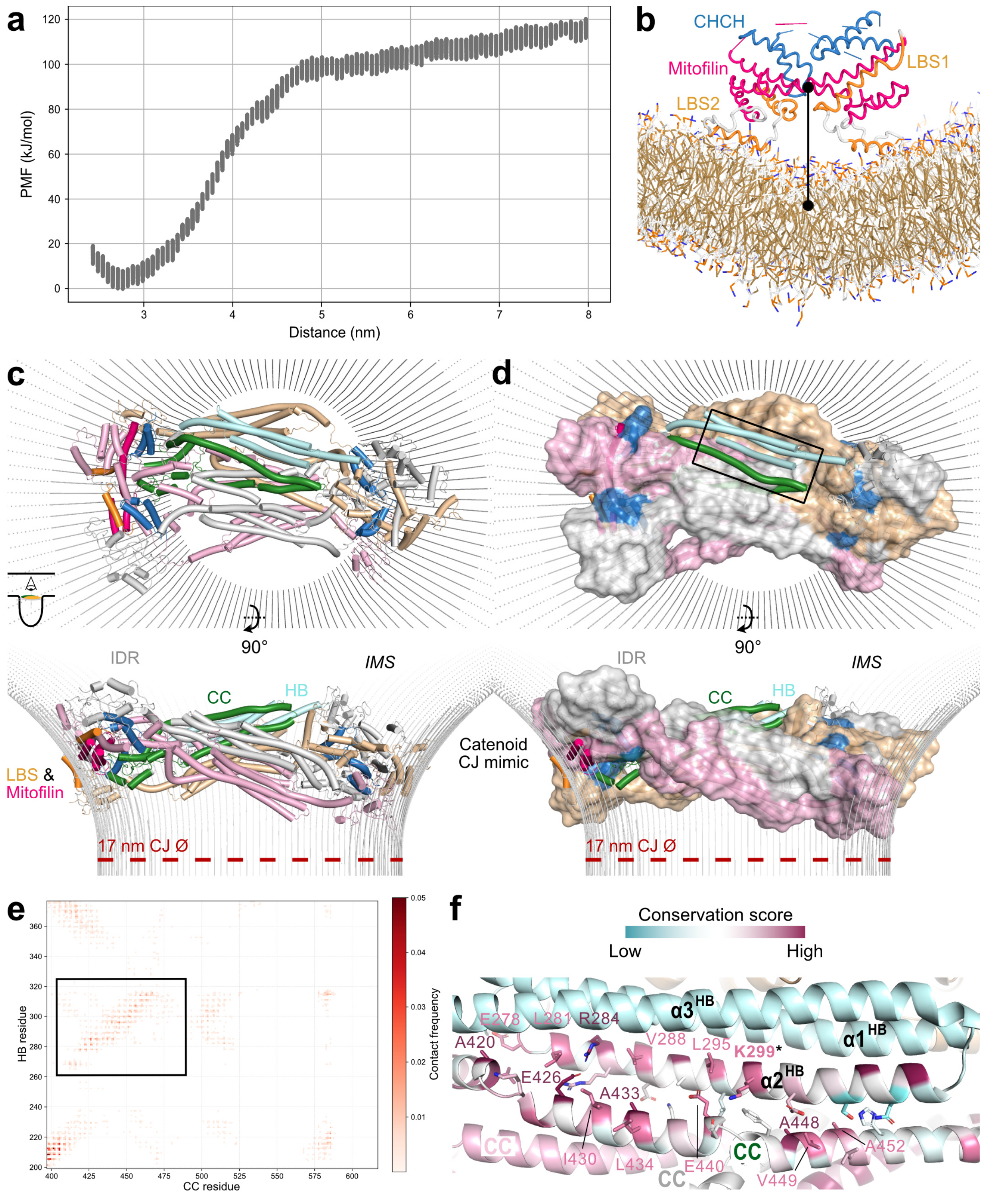

Supplementary Figure 7

Supplementary Figure 7: IMM attachment experiments and all-atom MD simulations for human Mic60-Mic19 on a CJ mimic

a Potential-mean-force (PMF) plot of the interaction of the C-terminal mitofilin-CHCH lobe to an IMM mimic. A release of approximately 100 kJ/mol was calculated upon binding. **b** Cartoon illustration of the interaction of the C-terminal mitofilin domain dimer (containing the mitofilin and the CHCH domains) to an IMM mimic. Black circles indicate the centre of each object, while a black bar indicates their distance, as plotted in a.

c, d Final snapshots of the all-atom MD simulations after attachment to the catenoid, with the model represented as cylinders (**c**) or cylinders and surface (**d**). The base diameter of the catenoid, indicated in a red dashed line, is 17 nm. Note the compression of the IDRs in comparison to the Cα structure-based MD simulations shown in Fig. 2d. **e** Contact site analysis between the HB (y axis) and CC (x axis) of Mic60 after all-atom MD simulations, showing the global average across all chain combinations and all 30 all-atom trajectories. **f** Close-up of the largest intra-domain interface in Mic60 (> 3000 $Å^2$), marked with a box in **d** and **e**, centring on HB aa 278-311 and CC aa 416-456. Involved residues as identified in **e** are coloured by conservation score, as calculated by ConSurf for the full-length hsMic60 sequence[3], with highly conserved residues annotated. The disease-relevant residue K299 is marked with an asterisk.

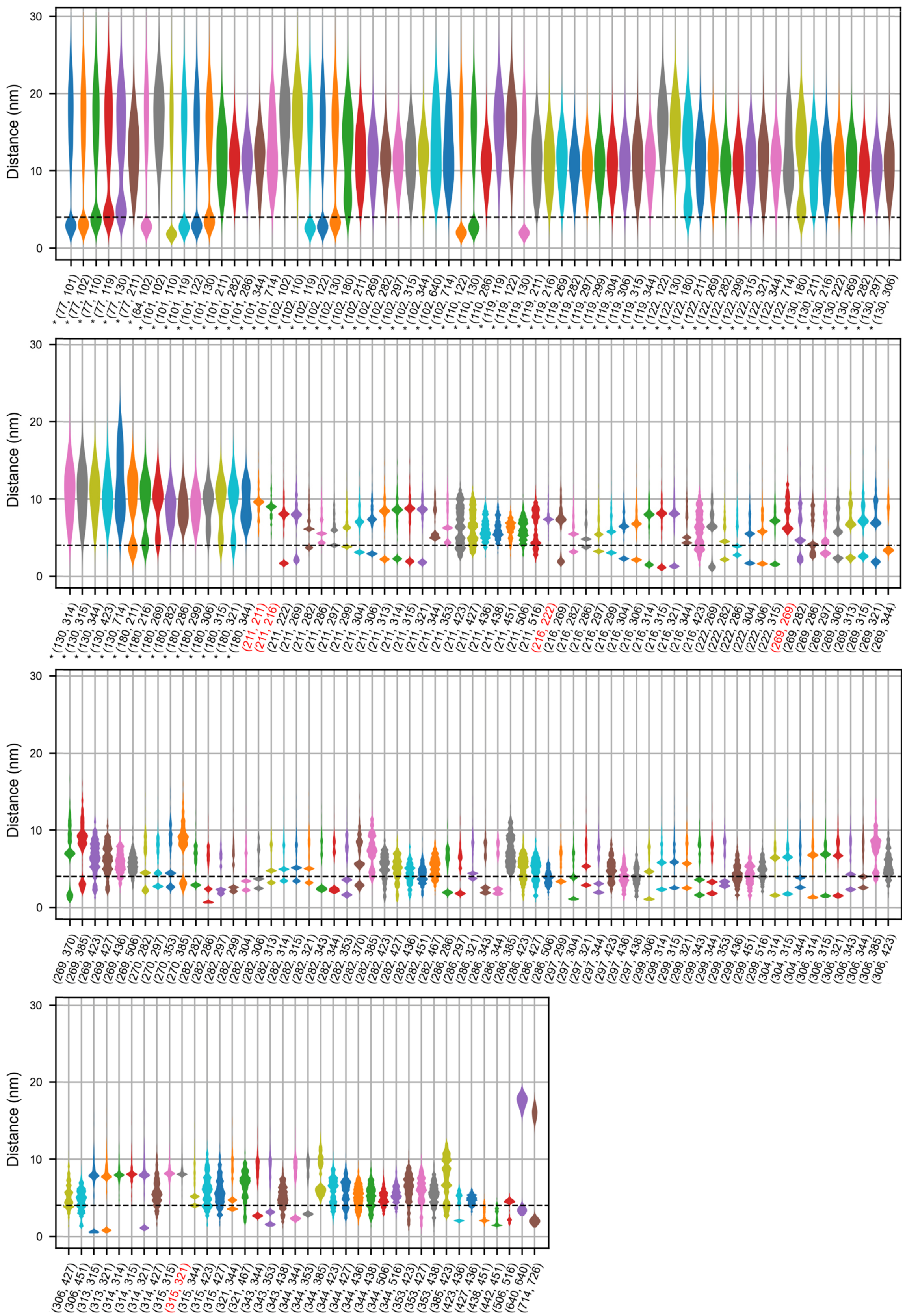

Supplementary Figure 8

Supplementary Figure 8: hsMic60 cross-linking dataset for MD validation

Violin plot of all 236 cross-links present in the dataset employing the enrichable and MS-cleavable cross-linker DSBSO from Zhu et al.[4]. The cross-link distance is plotted for each residue pair, including self-cross-links, across all Cα structure-based simulation frames. The threshold for validity was set at 4 nm (40 Å). Cross-links involving the IDR are marked with an asterisk. Cross-links that are not valid in any of the frames of the simulations are marked red. A table of the 236 residue-residue cross-links, their cross-link spectral matches and their n_scores from dataset PXD046382 is found in the Source data of this article under Fig. 3a.

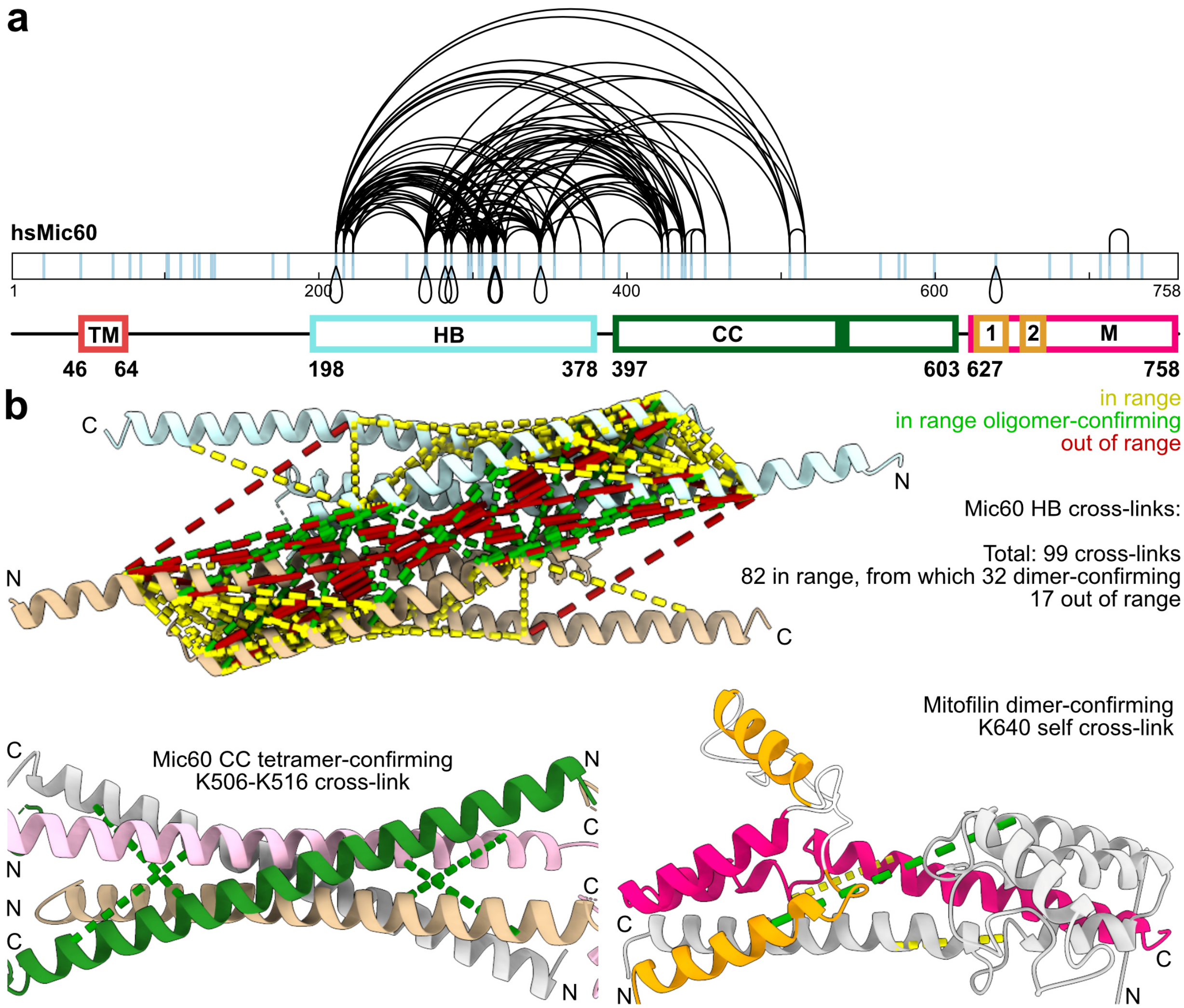

Supplementary Figure 9: *In organello* cross-linking as validation of the homology model prior to MD simulations

a Visualization of the DSBSO mitochondrial cross-linking dataset on the Mic60 sequence as in Fig. 3a, excluding the cross-links involving the IDR, generated with xiNET[5]. Blue bars across the protein sequence indicate the positions of lysines. **b** Analysis of the intra-domain cross-links of Mic60 before MD simulations. Cross-links under 40 Å are in range and coloured in yellow. The in range cross-links that confirm domain homo-oligomerization are coloured in green, and out of range cross-links are coloured in red. For the Mic60 HB dimer, 99 cross-links were detected (82 of them in range, with 32 of those confirming a HB dimer); for the Mic60 CC tetramer, one cross-link was detected and confirmed a homo-tetrameric assembly; for the mitofilin dimer, two cross-links were detected, one of which corresponded to a dimeric conformation.

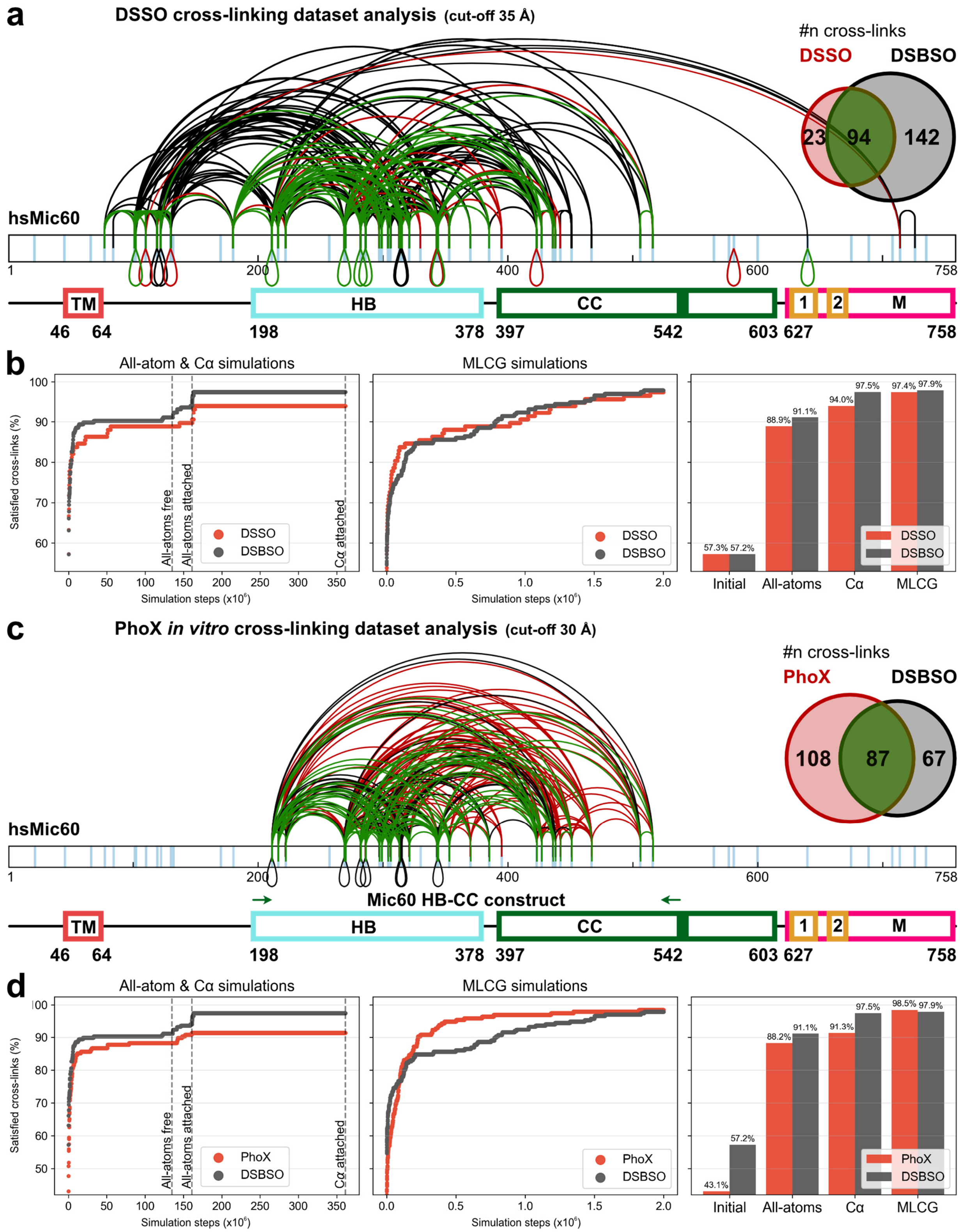

Supplementary Figure 10

Supplementary Figure 10: DSSO-based mitochondrial crosslinking dataset and *in vitro* cross-linking of a recombinantly expressed HB-CC construct

Comparison of the DSBSO-based mitochondrial crosslinking dataset from Zhu et al.[4] with (**a-b**) the DSSO-based mitochondrial cross-linking dataset from Zhu et al.[4], with 117 lysine-lysine cross-links, and (**c-d**) a PhoX-based *in vitro* cross-linking dataset from a recombinantly expressed HB-CC construct (hsMic60 aa 198-542), with 195 lysine-lysine cross-links.

a, c The cross-linking datasets are visualised on the Mic60 sequence as in Fig. 3a, generated with xiNET[5]. Blue bars across the protein sequence indicate the positions of lysines. Arrows indicate the construct boundaries for the recombinant construct. A Venn diagram illustrates the total number of cross-links for each cross-linker (red for DSSO (**a**) or PhoX (**c**), black for DSBSO) and their overlap (green), with cross-links coloured accordingly in the visualization on the Mic60 sequence. **b, d** The percentage of cross-links satisfied across the frames of the simulation, calculated for DSSO (**b**) or PhoX (**d**) (red) and plotted as for the DSBSO mitochondrial dataset (black, see also Fig. 3b). Note that the recombinantly expressed HB-CC construct does not contain any IDR residues, so no “excluding IDR” graphs are presented. Cut-off was set to 35 Å for DSSO[4] and 30 Å for PhoX[6].

Supplementary Table 1: Data collection and refinement statistics (molecular replacement)

	Mic60 HB (PDB ID: 9QWR)	*Re-processed with 2.95 Å resolution cut-off*
**Data collection**		
Beamline	BESSY 14.1	
Wavelength (Å)	0.9184	
Space group	$P4_12_12$ (92)	
Cell dimensions		
a, *b*, *c* (Å)	76.12 76.12 168.41	
α, β, γ (°)	90.0 90.0 90.0	
Resolution (Å)*	33.68 - 2.78 (2.95 - 2.78)	33.68 - 2.95 (3.13 – 2.95)
R_{meas} (%)	13.7 (324.2)	11.7 (170.1)
R_{pim} (%)	4.2 (118.7)	4.8 (72.2)
$CC_{1/2}$ (%)	99.9 (30.1)	99.9 (64.9)
I / σI	12.48 (0.67)	14.99 (1.49)
Completeness (%)	99.6 (97.4)	99.5 (98.1)
Redundancy	11.4 (11.5)	10.06 (10.56)
**Refinement**		
R_{work} / R_{free} (%)	24.5 / 28.3 (38.9 / 44.9)	22.8 / 27.9 (34.3 / 39.9)
Reflections	13,089 (2,028)	11,014 (1,065)
No. atoms		
Protein	2,542	2,542
Water	1	1
B-factor		
Protein	94.35	94.45
R.m.s. deviations		
Bond lengths (Å)	0.003	0.008
Bond angles (°)	0.48	0.90
Ramachandran Plot		
Outliers (%)	0.00	0.00
Allowed (%)	0.62	1.55
Favoured (%)	99.38	98.45
Rotamer outliers (%)	0.75	6.39
Clashscore	5.23	6.01

*Values in parentheses are for the highest-resolution shell. R_{pim} was calculated using AIMLESS in the CCP4i2 suite[7,8]. Correlation coefficient between two independent subsets ($CC_{1/2}$) according to Karplus and Diederichs[9]. Model geometry according to Molprobity[10]. The resolution cut-off in the left column was chosen along the assumption that data with $CC_{1/2}$ values down to 0.1–0.2 still contain valid, non-random signal that improves structural refinement[9]. In the right column, a more conservative resolution cut-off was chosen.

Supplementary Table 2: Molecular dynamics system setups

System	Total atoms	Water molecules (atoms)
All-atom main model	799,560	251,128 (753,384)
Cα main model	2,936	0
MLCG transferable main model	14,604	0
All-atom alternative model	1,991,085	648,303 (1,944,909)
MARTINI C-terminal lobe binding to IMM	117,141	95,091 (95,091)

References of the Supplementary Information

1. van Kempen, M. *et al.* Fast and accurate protein structure search with Foldseek. *Nature Biotechnology* **42**, 243-246 (2024). https://doi.org/10.1038/s41587-023-01773-0
2. McGibbon, R. T. *et al.* MDTraj: A Modern Open Library for the Analysis of Molecular Dynamics Trajectories. *Biophys J* **109**, 1528-1532 (2015). https://doi.org/10.1016/j.bpj.2015.08.015
3. Yariv, B. *et al.* Using evolutionary data to make sense of macromolecules with a “face-lifted” ConSurf. *Protein Science* **32**, e4582 (2023). https://doi.org/https://doi.org/10.1002/pro.4582
4. Zhu, Y. *et al.* Cross-link assisted spatial proteomics to map sub-organelle proteomes and membrane protein topologies. *Nat Commun* **15**, 3290 (2024). https://doi.org/10.1038/s41467-024-47569-x
5. Combe, C. W., Fischer, L. & Rappsilber, J. xiNET: cross-link network maps with residue resolution. *Mol Cell Proteomics* **14**, 1137-1147 (2015). https://doi.org/10.1074/mcp.O114.042259
6. Steigenberger, B., Pieters, R. J., Heck, A. J. R. & Scheltema, R. A. PhoX: An IMAC-Enrichable Cross-Linking Reagent. *ACS Central Science* **5**, 1514-1522 (2019). https://doi.org/10.1021/acscentsci.9b00416
7. Evans, P. R. & Murshudov, G. N. How good are my data and what is the resolution? *Acta Crystallogr D Biol Crystallogr* **69**, 1204-1214 (2013). https://doi.org/10.1107/s0907444913000061
8. Potterton, L. *et al.* CCP4i2: the new graphical user interface to the CCP4 program suite. *Acta Crystallographica Section D* **74**, 68-84 (2018). https://doi.org/doi:10.1107/S2059798317016035
9. Karplus, P. A. & Diederichs, K. Linking crystallographic model and data quality. *Science* **336**, 1030-1033 (2012). https://doi.org/10.1126/science.1218231
10. Williams, C. J. *et al.* MolProbity: More and better reference data for improved all-atom structure validation. *Protein Science* **27**, 293-315 (2018). https://doi.org/https://doi.org/10.1002/pro.3330
