# Supplementary material for: Integrative structural modelling reveals the human Mic60-Mic19 subcomplex as a diffusion barrier in mitochondria
Source: Nat Commun. 2026 Sep 26;17:10234. doi: 10.1038/s41467-026-77869-3 (PMC13615979; doi:10.1038/s41467-026-77869-3)
Supplement: Supplementary file 2 [file 41467_2026_77869_MOESM2_ESM.pdf]

nature portfolio

Peer Review File

Integrative structural modelling reveals the human Mic60-Mic19 subcomplex as a diffusion barrier in mitochondria

Corresponding Author: Professor Oliver Daumke

This file contains all reviewer reports in order by version, followed by all author rebuttals in order by version.

Version 0:

Reviewer comments:

Reviewer #1

(Remarks to the Author)
In this study E. Nathanail et al. determined the crystal structure of Mic60 and performed molecular dynamic simulations with the human Mic60-Mic19 subcomplex of MICOS. This structure was validated with previously published cross-linking data. The study is well described and combines several techniques, resulting in a comprehensive evaluation of the complex and computational characterization of its function as a diffusion barrier. Based on my own expertise this review is mostly focused on the use of the crosslinking data in the study.
Several points should be addressed before publishing this manuscript.
The authors use cross-linking data to validate the structural models. The original dataset contains triplicate data with DSSO and monoplicate data with DSBSO, yet the authors choose DSBSO. This is an unexpected choice of linker since structural modeling typically benefits from a shorter spacer arm of the cross-linking reagent (~10.1 Å for DSSO vs ~14 Å DSBSO). The length of the spacer arm, combined with the generous 40 Å distance constraints allow for a higher percentage of the cross-links overfitting with the model. I suggest this to be discussed in the manuscript. Since both are Lysine-Lysine linkers, are the cross-links that fit the model also validated by the DSSO data? To provide a clear view of the cross-link distances fitted to the model, I suggest adding a plot with the number of cross-links fitted and the distance, and indicating if they are validated by the DSSO data.

In the text, it is unclear whether they refer to "unique cross-links" as peptide-peptide or residue-residue unique links. Additionally, it would be valuable to show by how many cross-link spectral matches these unique links are supported since the cross-linking experiment was done in monoplicate.
A known issue in XL-MS for structural modelling when dealing with multimeric structures is the lack of information about which of the multimer subunits the cross-link is connected. Has this been taken into account for plotting the cross-links in the structure? Was the cutoff distance sufficient to deal with this?

Other comments:
1. In all the gels, write units and label the molecular weight estimations next to the gels.
2. In Figure 3A add the residue numbers in the protein sequence for each of the Mic60 domains.
3. In Figure 3C there are links in the bottom part of the figure that were cropped; this makes the interpretation of the figure unclear.
4. In line 761, there is a typo: "The trajectory that satisfied the highest number of cross-linkers was selected for

Reviewer #2

(Remarks to the Author)
Thank-you for the opportunity to review the manuscript by Nathanail et al.

The paper describes the determination of the structure of the helical bundle domain of the Mic60 (Mic60 HB) protein from humans and the formulation of a model of the Mic60-Mic19 complex that forms part of the mitochondrial contact site and cristae organising system (MICOS) assembly. MICOS is essential for forming and stabilising cristae junctions, which are crucial for proper mitochondrial function.

The structure of the Mic60 HB is well determined, although I would like the authors to revisit the applied resolution cut-off for

the diffraction data and to add additional statistics to Table S1. Specifically, the I/sigI and CC1/2 are too low in the highest bin I think – the inclusion of the overall Rpim and Rfactors for refinement in the highest resolution bins would help determine the appropriate resolution for the data.

The structure shows a homo dimer and the authors carry out a beautiful set of experiments to establish that the dimer in the crystal represents the oligomeric state of the protein in solution.

The second part of the paper is the modelling of the Mic60-Mic19 complex – built on crystal structures of Mic60 HB and other domains from non-human sources. The authors then follow with MD to refine this model and align it with existing cross linking data.

My expertise is not in modelling/MD, so I cannot comment on the technical aspects of the model generation. However, I do feel that for publication in this journal, the model should be accompanied by extensive experimental data for its validation. I realise the authors draw on a cross linking data base, but I do not feel this is sufficient. Experiments such as those carried out to validate the Mic60 HB dimer either with recombinant proteins: eg. engineering disulfide bonds at relevant interfaces; cross linking experiments with mass spectrometric analysis, etc should accompany this model. Alternatively using KO cell lines and recomplimentation with mutations that are designed to disrupt protein-protein interfaces or similar could strengthen the case for this model. Finally I am wondering whether recent cryo-ET studies of intact mitochondria might assist with validation?

I realise these are challenging studies, but for publication at this level I feel they are required.

Reviewer #3

(Remarks to the Author)
The manuscript describes an integrative structural modeling approach to the human Mic60-Mic19 subcomplex, which constitutes the mitochondrial cristae junctions. The complex structure was modeled by using newly solved dimeric helical bundle structures, which were optimized through atomistic and coarse-grained molecular dynamics (MD) simulations. The optimized complex structures were validated by in organello cross-linking data. The substantial efforts led to a reliable structural model that would function as a diffusion barrier at the cristae junctions. Some issues in the simulation parts to be addressed are listed below.

1) MD simulations suggest HB-CC packing, which was validated by cross-linking data. What kind of physical interactions drive the packing, and which residues are mainly involved in the process?
2) Figure 3B shows that MLCG has the best agreement among all-atom and Ca, although it is described that it “led to an artificial compression of the IDRs” (p 11, L 266). Does that mean that the IDRs should be compact to be consistent with the data?
3) Because the current model excludes Mic19 parts other than the CHCH domain, it should be discussed how it affects the presented results, such as permeability.

Minor point:
Page 4, Line 658: “hydrogen mass was set to 4 atomic mass units (amu) for stability.” It should be noted that this is for a large timestep.

Version 1:

Reviewer comments:

Reviewer #1

(Remarks to the Author)
the authors have addressed my issues to satisfaction

Reviewer #2

(Remarks to the Author)
The authors have addressed my comments generally. I am pleased to see experimental validation of the model now included.

I still however do not agree with the applied resolution cut-off for the diffraction data. In my view, the CC1/2 and I/sigI are too low in the highest resolution bin. The R and Rfree values for the refinement are too high in this bin also. For publication, the resolution should be adjusted lower so the CC1/2 is at least 0.5 in the highest bin and the R/Rfree values following refinement are more reasonable.

Reviewer #3

(Remarks to the Author)

The authors sufficiently addressed my concerns. I believe it is ready for publication.

Open Access This Peer Review File is licensed under a Creative Commons Attribution 4.0 International License, which permits use, sharing, adaptation, distribution and reproduction in any medium or format, as long as you give appropriate credit to the original author(s) and the source, provide a link to the Creative Commons license, and indicate if changes were made.
In cases where reviewers are anonymous, credit should be given to 'Anonymous Referee' and the source.
The images or other third party material in this Peer Review File are included in the article's Creative Commons license, unless indicated otherwise in a credit line to the material. If material is not included in the article's Creative Commons license and your intended use is not permitted by statutory regulation or exceeds the permitted use, you will need to obtain permission directly from the copyright holder.
To view a copy of this license, visit https://creativecommons.org/licenses/by/4.0/

RESPONSE TO REVIEWERS' COMMENTS

We would like to thank the three reviewers for their very constructive feedback. We sought to address their concerns by conducting new experiments and modifying main text, figures and figure legends, as indicated in our detailed point-by-point response below. We believe that the manuscript has greatly benefitted from these modifications.

Reviewer #1 (Remarks to the Author):
In this study E. Nathanail et al. determined the crystal structure of Mic60 and performed molecular dynamic simulations with the human Mic60-Mic19 subcomplex of MICOS. This structure was validated with previously published cross-linking data.
The study is well described and combines several techniques, resulting in a comprehensive evaluation of the complex and computational characterization of its function as a diffusion barrier. Based on my own expertise this review is mostly focused on the use of the crosslinking data in the study.
Thank you very much!

Several points should be addressed before publishing this manuscript.
The authors use cross-linking data to validate the structural models. The original dataset contains triplicate data with DSSO and monoplicate data with DSBSO, yet the authors choose DSBSO. This is an unexpected choice of linker since structural modeling typically benefits from a shorter spacer arm of the cross-linking reagent (~10.1 Å for DSSO vs ~14 Å DSBSO).
We appreciate the concerns and agree that also the DSSO data possess powerful potential for structural analysis, especially because of its shorter linker arm as the reviewer highlights. Indeed, we had compared the DSSO and DSBSO datasets already in detail prior to our first submission. To illustrate why we chose to highlight the DSBSO dataset, we have now included also the DSSO data in Supplementary Fig. 10A and B and explained our rationale in the main text. Since DSBSO is an enrichable cross-linker, this dataset contains more residue-residue cross-links for Mic60 (236 compared to 117 for DSSO) with a better average cross-link spectral match number (a confidence score for assigning a specific, unique cross-linked peptide pair, e.g. 7.3 compared to 2.6). For clarity, we have now added the DSSO dataset residue-residue cross-links, their cross-link spectral matches and their n_scores from dataset PXD032132 in the Source data of Supplementary Fig. 10A. The equivalent data for the DSBSO dataset can be found in the Source Data of Fig. 3. See also next comment.

The length of the spacer arm, combined with the generous 40 Å distance constraints allow for a higher percentage of the cross-links overfitting with the model. I suggest this to be discussed in the manuscript. Since both are Lysine-Lysine linkers, are the cross-links that fit the model also validated by the DSSO data? To provide a clear view of the cross-link distances fitted to the model, I suggest adding a plot with the number of cross-links fitted and the distance, and indicating if they are validated by the DSSO data.
Thank you. Based on this concern, we have included a comparison of the DSSO and DSBSO dataset in the Results section and in the Methods section. We studied satisfaction rates of the DSSO dataset on our model, using a 35 Å distance cut-off, which is 5 Å shorter than the original 40 Å cut-off described by Zhu et al. (PMID: 38632225) and from the DSBSO cross-linker, as indicated by the reviewer in the above comment. To this end, we applied the same pipeline as for the DSBSO dataset (see modified Methods). By visualizing the specific links using xiNet, we observe that the vast majority of DSSO residue-residue cross-links are also found in the DSBSO dataset (94 of 117 cross-links). In MD simulations the DSSO data reach a high cross-link satisfaction rate of up to 94% after Cα and 97% after MLCG simulations. In summary, the DSSO dataset recapitulates the findings of the DSBSO dataset, even with its shorter cross-linking distance restraint of 35 Å. We added Supplementary Figs. 10A, B. As requested, we indicated which crosslinks are present and/or validated in the DSSO vs DSBSO data in the Source Data of Supplementary Fig. 10A. The modified result section now reads:

To probe the data with a more stringent lysine-to-lysine distance, we compared the DSBSO-based dataset with a disuccinimidyl sulfoxide (DSSO)-based dataset from the same publication (Zhu et al., 2024). DSSO is a shorter crosslinker compared to DSBSO and, accordingly, allows

for a shorter, more stringent distance cut-off of 35 Å. However, DSSO yields fewer and less confident cross-links compared to DSBSO as it is not enrichable (see Source data). The comparison revealed that the majority of DSSO residue-residue cross-links were recapitulated in the DSBSO dataset (Supplementary Fig. 10A). Furthermore, the DSSO-based residue-residue cross-links show a high satisfaction rate of 94% across the MD simulations, which is consistent with our DSBSO analysis even under more stringent cross-linking distances (Supplementary Fig. 10B).

In the text, it is unclear whether they refer to "unique cross-links" as peptide-peptide or residue-residue unique links. Additionally, it would be valuable to show by how many cross-link spectral matches these unique links are supported since the cross-linking experiment was done in monoplicate.
We have corrected the text to indicate that unique cross-links are unique residue-residue links, see modified Results:

The database contains 236 unique residue pairs cross-links for hsMic60 distributed across the sequence (Fig. 3A, Supplementary Fig. 8, see also Source Data for a detailed overview).

We have also added a table of the 236 residue-residue cross-links from the DSBSO data, their cross-link spectral matches and their n_scores from dataset PXD046382 in the Source Data of Fig. 3A.

A known issue in XL-MS for structural modelling when dealing with multimeric structures is the lack of information about which of the multimer subunits the cross-link is connected. Has this been taken into account for plotting the cross-links in the structure? Was the cutoff distance sufficient to deal with this?
To clarify this point, we expanded the method section and also added more explanation to the corresponding figure legend. In brief, we used ResPairs and "Find shortest" to identify the most proximal possible cross-linked subunit. To account for the multiplicity of chains, we considered all possible crosslinks (16 possibilities for residue-residue and six for self cross-links) and plotted the distance distribution plots across simulation frames as violin plots in the Supplementary Fig. 8. When visualizing on Chimera and XMAS, we chose the option "Find shortest" to only show the most proximal cross-link for ease of illustration. The corresponding method section reads now:

Due to the multimeric assembly of the Mic60-Mic19 complex, there were 16 possibilities for residue-residue cross-links and six possibilities for self cross-links. We used ResPairs and "Find shortest" in ChimeraX to identify the most proximal possible cross-linked subunit. To account for the multiplicity of chains, we considered all possible crosslinks and plotted their distance distribution across simulation frames as violin plots in the Supplementary Fig. 8. The cross-link satisfaction percentage was calculated two-fold. Firstly, for individual experimental set-ups, it was calculated cumulatively across all simulation frames and considered satisfied when two residues of any chain fulfilled the distance cut-off for at least 1% of frames at any time up to the indicated timepoint. This provided better insight into the overall proximity of the lysine pairs throughout the simulations. Secondly, for comparing different set-ups, such as the preferred model with the alternative model or with the MLCG simulations, cross-link satisfaction was calculated, at a given time, in consideration of the mean and standard deviation of the lysine distance over the independent runs, to account for the differences in the number of independent trajectories per experimental set-up.

Other comments:
1. In all the gels, write units and label the molecular weight estimations next to the gels.
This was corrected, as requested.

2. In Figure 3A add the residue numbers in the protein sequence for each of the Mic60 domains.
Corrected, as requested, also for Supplementary Fig. 9.

3. In Figure 3C there are links in the bottom part of the figure that were cropped; this makes the interpretation of the figure unclear.
Thanks, we have included an uncropped image of Fig. 3C in the modified version.

4. In line 761, there is a typo: “The trajectory that satisfied the highest number of cross-linkers was selected for
Thanks, corrected.

Reviewer #2 (Remarks to the Author):

Thank-you for the opportunity to review the manuscript by Nathanail et al.

The paper describes the determination of the structure of the helical bundle domain of the Mic60 (Mic60 HB) protein from humans and the formulation of a model of the Mic60-Mic19 complex that forms part of the mitochondrial contact site and cristae organising system (MICOS) assembly. MICOS is essential for forming and stabilising cristae junctions, which are crucial for proper mitochondrial function.

The structure of the Mic60 HB is well determined, although I would like the authors to revisit the applied resolution cut-off for the diffraction data and to add additional statistics to Table S1. Specifically, the I/sigI and CC1/2 are too low in the highest bin I think – the inclusion of the overall Rpim and Rfactors for refinement in the highest resolution bins would help determine the appropriate resolution for the data.
Thank you very much! As requested, we now provide the overall R_{pim} and the R_{meas} in the data collection table (Table 1). To determine the resolution cut-off of the dataset in the most objective way, we used the standard settings of XDSapp, which defines the value in an iterative approach using the significance of half-dataset correlation CC(1/2) in the highest resolution shell, according to Karplus and Dietrichs (PMID 22628654). We included this information in the method section.
The pdb and mmCIF files have already been released under PDB ID 9qwr (https://www.rcsb.org/structure/9QWR) so that the reviewers can directly evaluate the quality of our structural data.

The structure shows a homo dimer and the authors carry out a beautiful set of experiments to establish that the dimer in the crystal represents the oligomeric state of the protein in solution.
Thank you very much!

The second part of the paper is the modelling of the Mic60-Mic19 complex – built on crystal structures of Mic60 HB and other domains from non-human sources. The authors then follow with MD to refine this model and align it with existing cross linking data.

My expertise is not in modelling/MD, so I cannot comment on the technical aspects of the model generation. However, I do feel that for publication in this journal, the model should be accompanied by extensive experimental data for its validation. I realize the authors draw on a cross linking data base, but I do not feel this is sufficient. Experiments such as those carried out to validate the Mic60 HB dimer either with recombinant proteins: eg. engineering disulfide bonds at relevant interfaces; cross linking experiments with mass spectrometric analysis, etc should accompany this model.
Thank you very much for the suggestion. To further validate our structural model and the cross-link data obtained from isolated mitochondria, we now also characterize the assembly of a recombinantly expressed, extended Mic60 construct, including the HB and adjacent coiled-coil (hsMic60 aa 198-542, for purification see Methods lines 535-544). According to our simulations, this region forms the core of the MICOS complex, and it also contains most cross-links of the folded Mic60 domains (153 out of 236 DSBSO cross-links or 65%, rising up to 99% when excluding the IDR).
We cross-linked the purified construct *in vitro* with the short cross-linker PhoX (as indicated in an entirely new paragraph in the Methods). Indeed, we observed that the *in vitro* cross-links recapitulate the same overall pattern of bridging the HB to the CC as the *in organello* cross-links (Supplementary Fig. 10C), but with a significantly shorter cut-off distance (30 Å for PhoX compared to 35 Å for DSSO and 40 Å for DSBSO). Thus, following the same procedure as for the *in organello* crosslinks, we observed that 91% of the *in vitro* cross-links were satisfied during the MD simulations, reaching up to 98% for the MLCG simulations. We have added the new Supplementary Figs 10C-D and updated the results section (see below). In addition, we

have uploaded the PhoX cross-linking data on the PRIDE repository. The modified Result section now reads:

To further validate the in organello cross-linking data and achieve higher spatial resolution, we recombinantly expressed a construct of the Mic60 HB and CC (hsMic60 residues 198-542, hereafter referred to as Mic60 HB-CC), which encompasses the majority of the cross-linked lysine residue pairs of the DSBSO dataset (65% for all, 99% excluding the IDR). We performed in vitro cross-linking using the short-armed cross-linker PhoX (Steigenberger et al., 2019) and observed that the same overall pattern of the DSBSO cross-links, in particular the bridging of the HB to the CC, is recapitulated in the purified construct (Supplementary Fig. 10C). Following the same procedure as for the in organello cross-links, we observed that 91% of the in vitro cross-links were satisfied at some point of the MD simulations, reaching up to 99% in the MLCG simulations, even using the more stringent cut-off of 30 Å of PhoX (Supplementary Fig. 10D). These in vitro experiments complement the in organello studies by validating the HB-CC packing in the core of our oligomeric model.

Alternatively using KO cell lines and recomplimentation with mutations that are designed to disrupt protein-protein interfaces or similar could strengthen the case for this model.
Thanks a lot for the suggestion. Indeed, we had already attempted to stably re-express MIC60 variants without the HB in two Mic60-deficient eukaryotic cell lines to characterize the assembly of Mic60 in the absence of the HB domain. However, despite several attempts and significant efforts, we were not able to isolate cell lines that stably re-expressed MIC60 and the HB deletion variant at or close to the endogenous expression levels. We could therefore not obtain conclusive data from these experiments.
In the meantime, a preprint appeared describing a detailed structure-function study of MIC60 including a specific domain deletion of the helical bundle (Rockfield et al, PMID 41889936). The authors found that re-expression of an Mic60 ΔHB variant in a Mic60 knockout cell line rescued basal and maximal respiration, and ATP production, and also restored assembly of the MICOS complex and cristae architecture, suggesting that the HB domain is largely dispensable for these functions. However, re-expression of this variant led to a fragmentation of the mitochondrial network, while other, possibly cell-type-specific roles of the HB in mitochondrial function, cannot be excluded. We now refer to this publication in our discussion (lines 453-459), which now reads:

A recent preprint (Rockfield et al., 2026) reported that re-expression of a MIC60 HB deletion variant in Mic60 knockout mouse embryonic fibroblasts restored assembly of the MICOS complex and rescued the Mic60 deletion phenotype in respiration and ATP production, suggesting that the HB domain is largely dispensable for these functions in fibroblasts; however, in the rescued cells, fragmentation of the mitochondrial network was observed while cristae morphology remained mostly intact (Rockfield et al., 2026). The latter finding is consistent with our evolutionary observation that the HB is not found in all MICOS- and CJ-possessing organisms. Instead, the HB may have distinct or cell-type-specific mitochondrial functions, for example to enlarge the volume occupied by the Mic60 CC in certain CJ morphologies or stress conditions. In particular, the packing of the HB onto the CC appears to be vital for Mic60 function in neurons, as residue K299 of the HB constitutes part of the interface, while the K299E mutation (895A>G) has been associated with optical neuropathy and developmental encephalopathy due to altered mitochondrial membrane architecture in human patients (Marco-Hernández et al., 2022).

Finally, I am wondering whether recent cryo-ET studies of intact mitochondria might assist with validation?
Indeed, cryo-ET approaches have recently been used to determine the structure of mitochondrial protein complexes, such as the oxidative phosphorylation machineries and ATP synthase, in their native environment. We have also intensively analyzed published and our own cryo-ET tomograms to obtain structural insights into the MICOS complex. However, so far, we were not able to identify MICOS as a prominent particle in the cryo electron tomograms,

despite its clearly defined localization at crista junctions. This is likely related to the relatively low mass of MICOS' soluble domains (~400 kDa) and its highly flexible architecture, as seen in our simulations. We have added this information to our discussion section, which now reads:

Experimental structure solution of the entire MICOS complex, for example, by single particle cryo electron microscopy or in situ by cryo electron tomography is challenging due to its relatively small yet highly flexible domain architecture, the complex' low copy number in CJs (Stephan et al., 2020), and the crowded CJ environment (Schuhmann et al., 2025). Compared to more static structures, the dynamic Mic60-Mic19 model proposed in this study rationalizes these challenges and may provide a more realistic structural representation required to understand MICOS function.

I realize these are challenging studies, but for publication at this level I feel they are required.

Reviewer #3 (Remarks to the Author):

The manuscript describes an integrative structural modeling approach to the human Mic60-Mic19 subcomplex, which constitutes the mitochondrial cristae junctions. The complex structure was modeled by using newly solved dimeric helical bundle structures, which were optimized through atomistic and coarse-grained molecular dynamics (MD) simulations. The optimized complex structures were validated by in organello cross-linking data. The substantial efforts led to a reliable structural model that would function as a diffusion barrier at the cristae junctions.
Thank you very much!

Some issues in the simulation parts to be addressed are listed below.

1) MD simulations suggest HB-CC packing, which was validated by cross-linking data. What kind of physical interactions drive the packing, and which residues are mainly involved in the process?
Thanks a lot for this question. We analyzed the HB-CC packing in more detail by determining the residues involved in this interface throughout our all-atom simulations (see modified Method section). In these analyses, we identified the interface between the HB and the CC as the most consistent and expansive, and studied its conservation in more detail using ConSurf. The residue pairs identified as statistically significant, as well as more information on their packing and conservation, were added to Supplementary Fig. 7E-F and the Results, which now reads:

AlphaFold2 predicted an extended helical connection between the Mic60 HB and the CC (see Materials and Methods), which consistently became sandwiched between these domains during the simulations (Supplementary Fig. 7E), forming a more compact core structure that covered almost the entire pore of the CJ. The folded core buries more than 3.000 $Å^2$ and is composed mainly of hydrophobic interactions framed by salt bridges between conserved residues of $α2^{HB}$ and the CC helical connection (Supplementary Fig. 7F). Accordingly, the HB-CC interaction remained stable throughout the simulation. In contrast, the IDRs were flexible in the coarse-grained Cα structure-based simulations, covering diverse trajectories within the remaining CJ space. In this way, the IDRs formed an extended network above and below the HB-CC core.

The interface involves surface-exposed residue Lys299, whose mutation is associated with encephalopathy and optical neuropathy. We included this information in the discussion (see also comments to reviewer 2).

Instead, the HB may have distinct or cell-type-specific mitochondrial functions, for example to enlarge the volume occupied by the Mic60 CC in certain CJ morphologies or stress conditions. In particular, the packing of the HB onto the CC appears to be vital for Mic60 function in neurons, as residue K299 of the HB constitutes part of the interface, while the K299E mutation (895A>G) has been associated with optical neuropathy and developmental encephalopathy due to altered mitochondrial membrane architecture in human patients (Marco-Hernández et al., 2022).

2) Fig. 3B shows that MLCG has the best agreement among all-atom and Cα, although it is described that it “led to an artificial compression of the IDRs” (p 11, L 266). Does that mean that the IDRs should be compact to be consistent with the data?
This statement is based on literature, since extensive MD simulation studies of IDRs have indicated that coarse-grained simulations better reflect their dynamic nature and minimize compression compared to all-atom MD simulations. We included this information in the Results:

Both the all-atom force field and the MLCG model led to an artificial compression of the IDRs,

as had been previously observed when simulating large IDR assemblies (Kasahara et al., 2019; Ruff et al., 2019; Shi et al., 2026) (Supplementary Figs. 7C, D). To more accurately capture IDR dynamics, the final frames from each of the 30 all-atom runs were therefore converted into coarse-grained Cα structure-based models (Clementi et al., 2000), leading to a refined final structure (Fig. 2D).

However, we were intrigued by the reviewer's question regarding the differences between the datasets, so we compared the MLCG and Cα-based simulations in more detail. We found that three cross-links of the DSBSO dataset were satisfied only in the MLCG and not in the Cα simulations (aa 211-211 within the IDR, 211-344 between IDR and HB and 269-269 within the HB), while two cross-links were valid only in Cα-based and not in the MLCG simulations (aa 101-344 and 102-344, both between the IDR and HB), indicating that the higher satisfaction rate is based on only one cross-link difference and not necessarily due to the IDR compression. We have included this information in the results section (lines 305-306) and provide the list of satisfied and unsatisfied cross-link in the Source data under Fig. 3A.

Notably, in both scenarios, meaning whether all cross-links were considered or those involving the IDR were excluded, MLCG simulations showed improved agreement with the experimental data at shorter simulation times compared to all-atom simulations and a similar agreement as the Cα-based structural simulations, differing by only net one cross-link (Fig. 3B, Supplementary Fig. 6C).

3) Because the current model excludes Mic19 parts other than the CHCH domain, it should be discussed how it affects the presented results, such as permeability.
Thanks, we had already included some limitation of our model in the discussion and we now specifically added the use of the truncated Mic19 construct (see Discussion 453-459).

It can be envisaged that the inclusion of further MICOS components into our model, most vitally the remaining folded domains of Mic19 or the membrane-embedded Mic10 subcomplex, will further restrict the size of particles that can cross the CJ pore. Furthermore, the Mic19 N-terminus has been proposed to reach over from the IMM to the OMM and contact the SAM complex (Tang et al., 2020), forming a flexible network contact site through dynamic movement, which could be included in future modelling studies.

Minor point:
Page 4, Line 658: "hydrogen mass was set to 4 atomic mass units (amu) for stability." It should be noted that this is for a large timestep.
Thank you for pointing it out, we adapted the methods as suggested.

Editorial requests:

* Please complete or update the following checklist to verify compliance with our research ethics and data reporting standards. Address all points on the checklist, revising your manuscript in response to the points if needed.
A reporting summary for this manuscript has been included in the revision.

DATA AND CODE AVAILABILITY
* All Nature Communications manuscripts must include a "Data Availability" section after the Methods section but before the References.
* We strongly encourage you to deposit all new data associated with the paper in a persistent repository where they can be freely and enduringly accessed. We recommend submitting the data to discipline-specific and community-recognised repositories;
A data availability statement including links to the pdb and cross-linking datasets has been included at the relevant position. No new code was reported in this manuscript.

* Official PDB validation reports must be provided with the resubmission. For cryoEM and X-ray crystallography data please upload the updated map and model files to the figshare repository integrated in our submission system (please see above for the information on figshare).
As requested, we provide updated validation reports with the manuscript. Furthermore, X-ray map and model files were uploaded in figshare (https://doi.org/10.6084/m9.figshare.32604771).

* To maximise the reproducibility of research data, we ask that you provide a Source Data file containing the raw data underlying the following types of display items:
- Any reported means/averages in box plots, bar charts, and tables
- Dot plots/scatter plots, especially when there are overlapping points
- Line graphs
- Uncropped and unprocessed scans of all blots and gels including all quantified replicates.
A source data file containing the relevant source data has been uploaded. The "Data Availability" section includes the statement "Source Data are provided with this paper."

* Please replace your bar graphs with plots that feature information about the distribution of the underlying data. All data points should be shown for plots with a sample size less than 10. For larger sample sizes, please consider box-and-whisker or violin plots as alternatives. Measures of centrality, dispersion and/or error bars should be plotted and described in the figure legend.
All relevant information is provided in relevant figures and also in the Source Data, as requested.

Add ORCIDs.
ORCIDs of all authors have been provided with the resubmission.
Evangelia Nathanail: https://orcid.org/0009-0004-9579-8074
Edoardo Rolando: https://orcid.org/0009-0001-8276-1406
Max Ruwolt: https://orcid.org/0009-0000-7220-406X
Iryna Zaporozhets: https://orcid.org/0000-0002-5846-3601
Fan Liu: https://orcid.org/0000-0002-2358-549X

Cecilia Clementi: https://orcid.org/0000-0001-9221-2358
Oliver Daumke: https://orcid.org/0000-0002-6190-1414

AUTHOR CHANGES ON REVISION

If there are any changes to the author list in the revised manuscript, please use this approval form www.nature.com/documents/nr-author-list-change-form.pdf, arranging for all authors on your paper to sign the statement confirming that they agree to the author list being changed, and add this document to your resubmission.

The author list has not been changed during the revision.

RESPONSE TO REVIEWERS' COMMENTS

Reviewer #1 (Remarks to the Author):
the authors have addressed my issues to satisfaction
Thank you very much!

Reviewer #2 (Remarks to the Author):

The authors have addressed my comments generally. I am pleased to see experimental validation of the model now included.
Thank you very much!

I still however do not agree with the applied resolution cut-off for the diffraction data. In my view, the CC1/2 and I/sigI are too low in the highest resolution bin. The R and Rfree values for the refinement are too high in this bin also. For publication, the resolution should be adjusted lower so the CC1/2 is at least 0.5 in the highest bin and the R/Rfree values following refinement are more reasonable.
To comply with this request, we have amended the data collection table (Supplementary Table 1) and added a column in which the X-ray data were re-processed with a more conservative resolution cut-off of 2.95 Å. As requested, the $CC_{1/2}$ is now > 0.5 in the highest resolution shell and the R_{work}/R_{free} factors are accordingly lower (38.9%/44.9% vs. 34.3%/39.9%), while the resulting models are virtually identical. We added a short note to the description of the table and mention the two resolution cutoffs values in the main manuscript.

Reviewer #3 (Remarks to the Author):

The authors sufficiently addressed my concerns. I believe it is ready for publication.
Thank you very much!
