# Supplementary material for: Integrative structural modelling reveals the human Mic60-Mic19 subcomplex as a diffusion barrier in mitochondria
Source: Nat Commun. 2026 Sep 26;17:10234. doi: 10.1038/s41467-026-77869-3 (PMC13615979; doi:10.1038/s41467-026-77869-3)
Supplement: Supplementary file 3 [file 41467_2026_77869_MOESM3_ESM.pdf]

Description of Additional Supplementary Files

Supplementary Video 1

Description: Video of all simulation frames of the Cα-based diffusion experiments of the human Mic60-Mic19 model in the catenoid. 200 spheres, here with the radius of 1.5 nm, were allowed to pass through the junction. There were no repulsion forces between the spheres, only between the spheres and the complex or the catenoid, allowing them all to be simulated simultaneously.
